# Reassessment of a juvenile *Daspletosaurus* from the Late Cretaceous of Alberta, Canada with implications for the identification of immature tyrannosaurids

**DOI:** 10.1038/s41598-019-53591-7

**Published:** 2019-11-28

**Authors:** Jared T. Voris, Darla K. Zelenitsky, François Therrien, Philip J. Currie

**Affiliations:** 1 University of Calgary, Department of Geoscience, Calgary, Alberta T2N 1N4 Canada; 2 Royal Tyrrell Museum of Palaeontology, Drumheller, Alberta T0J 0Y0 Canada; 3 University of Alberta, Biological Sciences, Edmonton, Alberta T6G 2R3 Canada

**Keywords:** Palaeontology, Taxonomy

## Abstract

*Daspletosaurus* is a large tyrannosaurine found in upper Campanian deposits of Alberta and Montana. Although several large subadult and adult individuals of this taxon are known, only one juvenile individual, TMP 1994.143.1, has been identified. This specimen has played a key role in the idea that juvenile tyrannosaurid individuals are difficult to differentiate among species. Here the taxonomic affinity of TMP 1994.143.1 is reassessed in light of a juvenile tyrannosaurine postorbital recently discovered in the Dinosaur Park Formation of Alberta. Anatomical comparisons and phylogenetic analyses reveal that TMP 1994.143.1 is referable to the albertosaurine *Gorgosaurus libratus*, whereas the new postorbital belongs to a small juvenile *Daspletosaurus*. This taxonomic reassignment of TMP 1994.143.1 results in the juvenile ontogenetic stage of *Daspletosaurus* being known only from two isolated cranial elements. The new postorbital provides insights into early *Daspletosaurus* ontogeny, revealing that the cornual process developed earlier or faster than in other tyrannosaurids. Although some ontogenetic changes in the postorbital are found to be unique to *Daspletosaurus*, overall changes are most consistent with those of other large tyrannosaurines. Our results also show that diagnostic features develop early in ontogeny, such that juveniles of different tyrannosaurid species are easier to differentiate than previously thought.

## Introduction

The discovery of juvenile specimens of tyrannosaurids in the past 30 years has greatly improved our understanding of the life history of these non-avian theropods^1–9^. Although some taxa are known from several individuals spanning a wide ontogenetic range, most are characterized by a paucity of specimens of early ontogenetic stages. Two of the best-represented tyrannosaurid species are the albertosaurine *Gorgosaurus libratus* and the tyrannosaurine *Daspletosaurus torosus*, sympatric taxa from the Upper Cretaceous (upper Campanian) Belly River Group of Alberta, Canada^10^. Whereas *Gorgosaurus* is represented by reasonably complete individuals ranging from small juveniles (~500 mm skull length) to large adults (>900 mm skull length), *Daspletosaurus* is known primarily from larger subadult and adult specimens (>880 mm skull length), with only a single juvenile individual reported (620 mm skull length)^4^.

**Figure 1:**
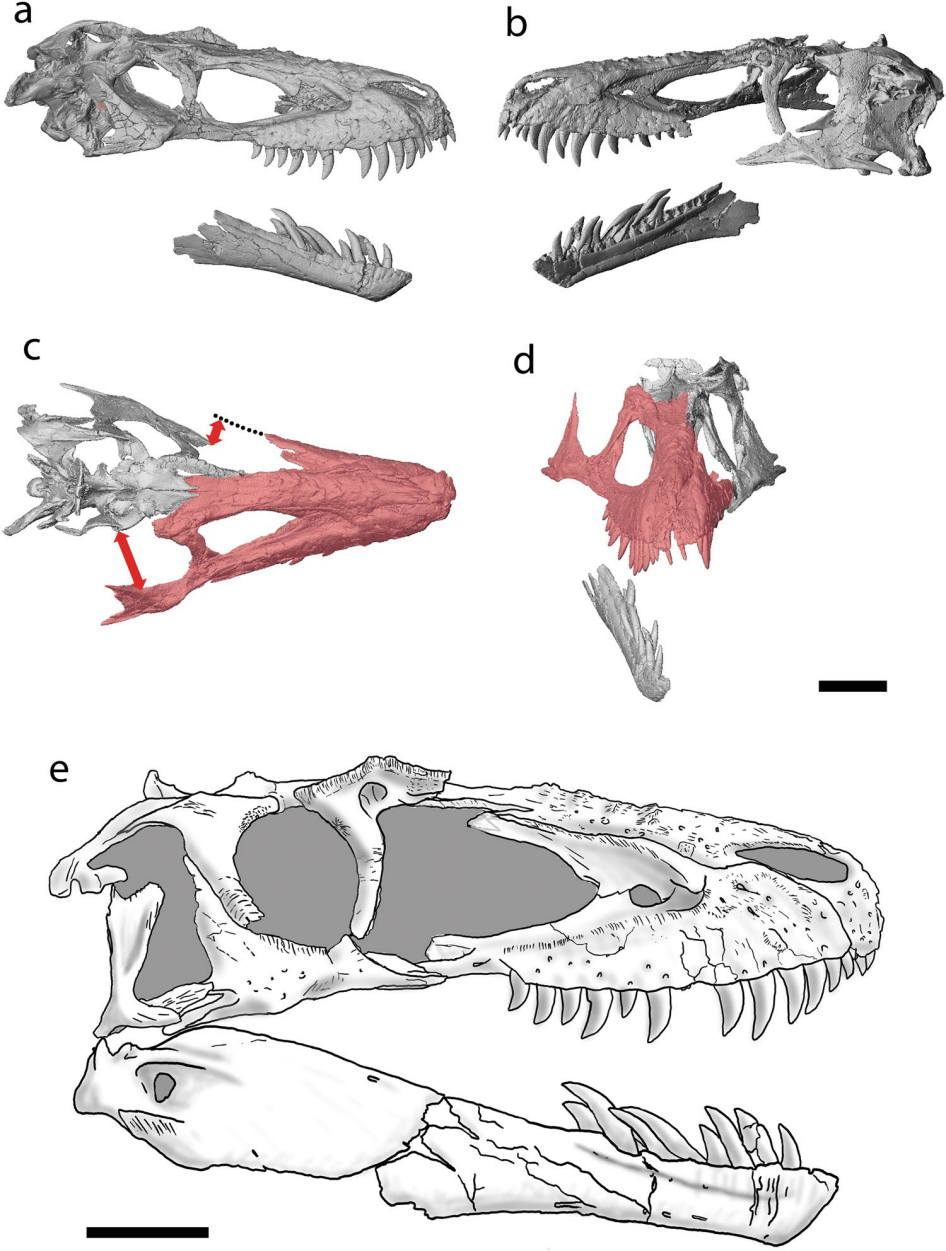
Skull reconstruction of TMP 1994.143.1. Digital rendering of skull based on CT data in right lateral view (**a**), left lateral view (**b**), dorsal view (**c**) and anterior view (**d**). Note that not all preserved elements were CT scanned. Skull reconstruction in right lateral view based on combination of preserved right and left elements (**e**). Digital renderings (**c**,**d**) show that the articulated bones of the rostrum (in red) has been mediolaterally widened due to taphonomic deformation, making the temporal region of the skull appear wide. Scale bars equal 100 mm. Institutional abbreviation: TMP, Royal Tyrrell Museum of Palaeontology.

As the only known juvenile skeleton of *Daspletosaurus*, TMP 1994.143.1 has long been a key specimen to help characterize the morphology of juvenile tyrannosaurines^4,9,11,12^ (Fig. 1). Studies have shown that TMP 1994.143.1 appears to share few features with *Daspletosaurus* (and other tyrannosaurines) but surprisingly many with albertosaurines^4,13^. These similarities may have played a large part in the idea that juveniles of albertosaurines and tyrannosaurines (and most tyrannosaurids) possess few diagnostic characteristics, thus rendering species difficult to differentiate among juveniles. However, as smaller tyrannosaurid individuals are known to possess apomorphies of their respective taxa^2,6–8,14,15^, it stands to reason that TMP 1994.143.1 should also possess diagnostic features of both *Daspletosaurus* and tyrannosaurines.

The recent discovery of a small tyrannosaurine postorbital, TMP 2013.18.11, which shares many similarities with adult *Daspletosaurus* specimens but differs significantly from TMP 1994.143.1, leads us to reassess the original taxonomic assignment of the purported juvenile *Daspletosaurus* specimen. Detailed anatomical examination and phylogenetic analyses of these and other tyrannosaurid specimens reveal that TMP 1994.143.1 is a juvenile-subadult *Gorgosaurus libratus*, whereas the small isolated postorbital TMP 2013.18.11 belongs to a juvenile *Daspletosaurus*. Although these results alter our understanding of *Daspletosaurus* ontogeny, they demonstrate that juvenile albertosaurines and tyrannosaurines, and probably juvenile tyrannosaurids in general, can be more readily differentiated than previously thought.

## Results

### Identification of TMP 2013.18.11

**Figure 2:**
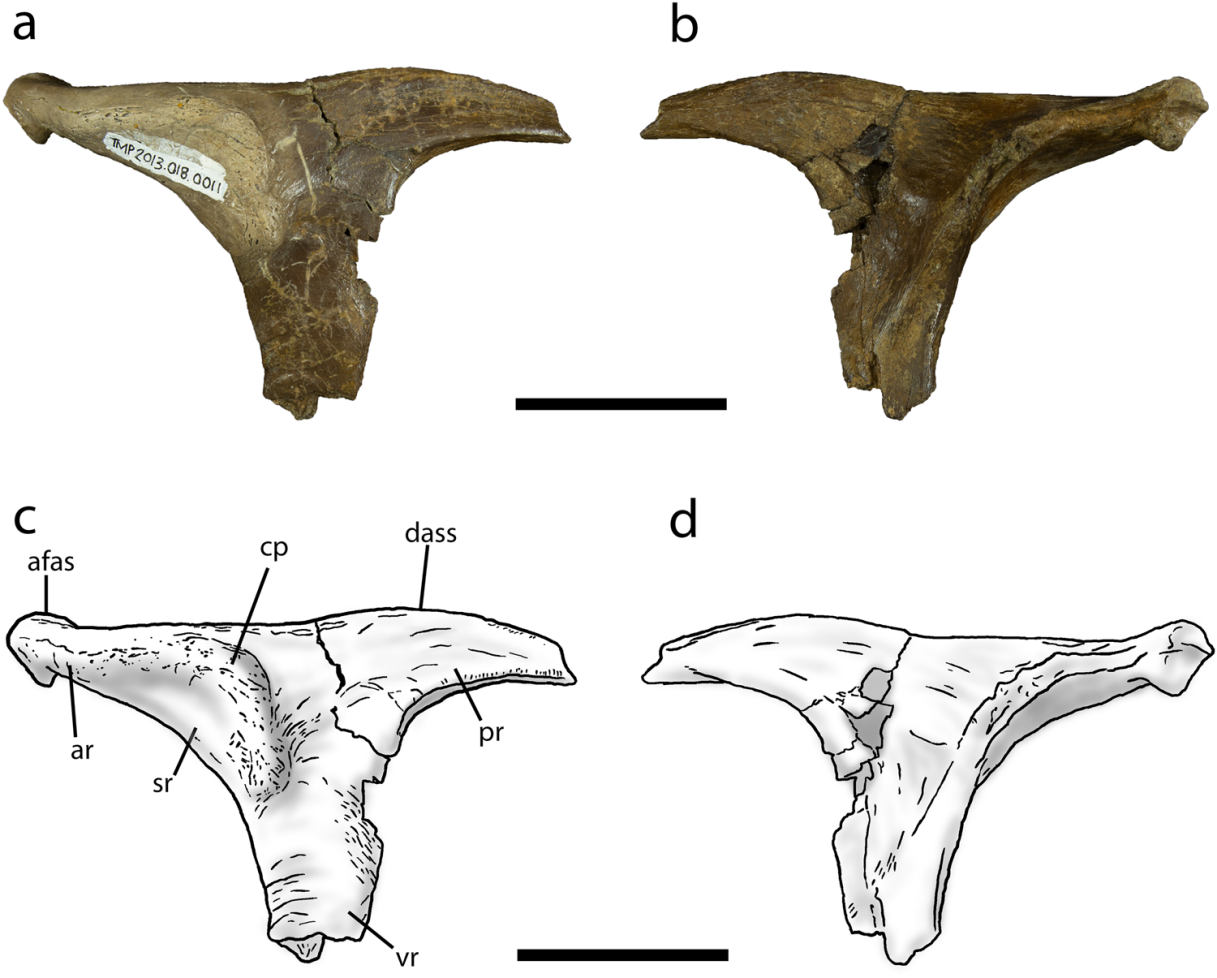
Juvenile *Daspletosaurus* postorbital TMP 2013.18.11 in lateral (**a**,**c**) and medial (**b**,**d**) views. Abbreviations: afas, anterior expansion of the frontal sutural surface; ar, anterior ramus; cp, cornual process; dass, anterior end of the dorsal contact for the squamosal; pr, posterior ramus; sr, smooth region separating cornual process from orbit margin; vr, ventral ramus. Scale bars equal 50 mm. Institutional abbreviation: TMP, Royal Tyrrell Museum of Palaeontology.

TMP 2013.18.11 is a small, isolated tyrannosaurid postorbital from the Dinosaur Park Formation of Alberta (Fig. 2). The specimen can be unambiguously identified as *Daspletosaurus* based on the presence of one autapomorphy of the genus: the dorsal articular surface for the squamosal extends to a point posterior to the anterior margin of the laterotemporal fenestra^9^. The specimen can also be identified as a tyrannosaurine, of which *Daspletosaurus* is the only known representative in the Dinosaur Park Formation, based on the presence of a distinct C-shaped cornual process that is separated from the orbital margin by a smooth bone surface^4,9,16–19^.

### Description of TMP 2013.18.11 and comparison to TMP 1994.143.1 and other tyrannosaurids

**Figure 3:**
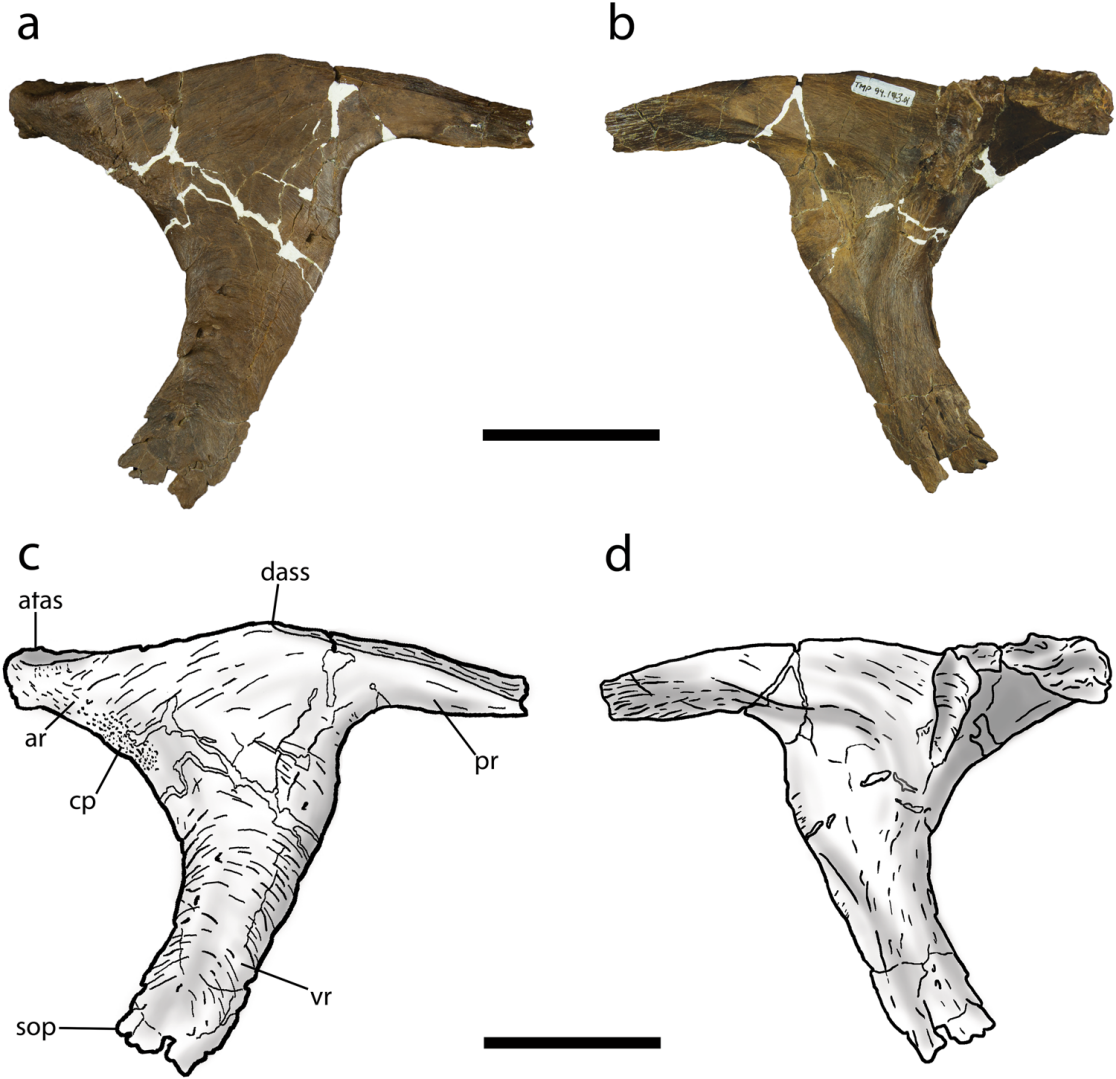
Left postorbital of TMP 1994.143.1 in lateral (**a**,**c**) and medial (**b**,**d**) views. Abbreviations as in Fig. 2 in addition to: sop, suborbital process. Scale bar equals 50 mm. Institutional abbreviation: TMP, Royal Tyrrell Museum of Palaeontology.

The small *Daspletosaurus* postorbital, TMP 2013.18.11, is well-preserved but is missing the laterosphenoid articular surface, the tip of the posterior ramus, and the distal end of the ventral ramus (Fig. 2). The element measures 118 mm in preserved length along the dorsal margin and is similar in size to the postorbital of a juvenile *Gorgosaurus* skull TMP 1986.144.1^2^, a specimen with an estimated skull length of 500 mm^4^. In comparison, the postorbital of TMP 1994.143.1 (skull length = 620 mm) measures 138 mm along the length of the dorsal bar (Fig. 3).

**Figure 4:**
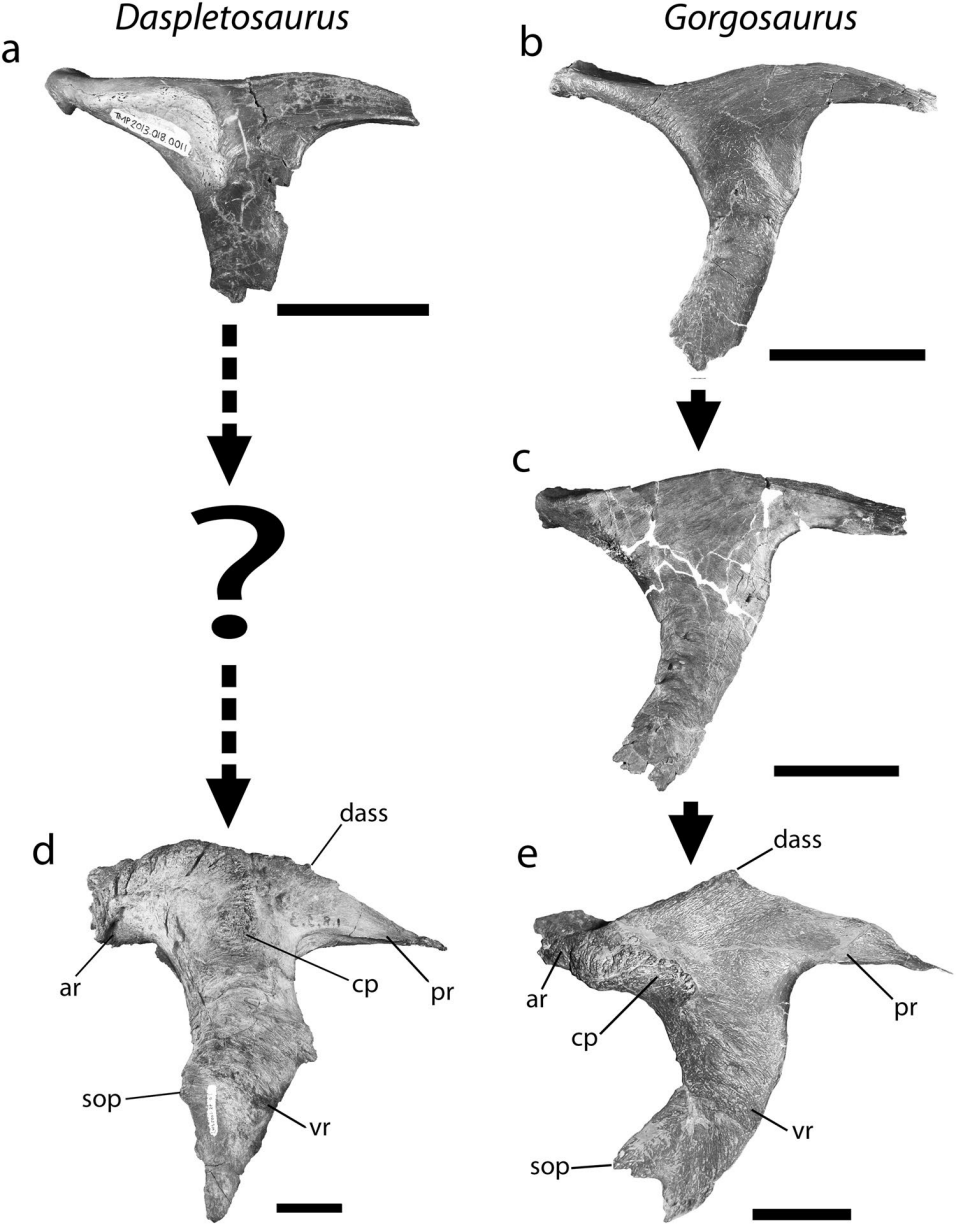
Growth series of the postorbital in *Daspletosaurus* (**a**,**d**) and *Gorgosaurus* (**b**,**c**,**e**). Elements shown in lateral view. (**a**) Small juvenile *Daspletosaurus* TMP 2013.18.11; (**b**) Small juvenile *Gorgosaurus* TMP 1986.144.1; (**c**) Large juvenile-subadult *Gorgosaurus* TMP 1994.143.1; (**d**) Adult *Daspletosaurus* TMP 2001.36.1; (**e**) Adult *Gorgosaurus* UALVP 10. The intermediate large juvenile-subadult *Daspletosaurus* condition is unknown. Abbreviations as in Figs. 2 and 3. Scale bars equal 50 mm. Institutional abbreviations: TMP, Royal Tyrrell Museum of Palaeontology; UALVP, University of Alberta Laboratory of Vertebrate Paleontology.

The morphology of the anterior ramus of the postorbital differs between TMP 2013.18.11 and TMP 1994.143.1. In TMP 2013.18.11, it is elongate and increases in depth proximally due to the presence of a swollen cornual process, giving it a triangular shape in lateral view (Figs. 2 and 4a). In adult *Daspletosaurus* specimens, the anterior ramus is short and deep, but rectangular in lateral view^4,9^ (Fig. 4d). In TMP 1994.143.1, the shape of the anterior ramus differs between the left and right elements, but both bear a stronger resemblance to those of albertosaurines than to *Daspletosaurus*. The anterior ramus is long and shallow on the right postorbital, as in juvenile *Gorgosaurus* specimens (TMP 1986.144.1, Fig. 4b), whereas it has a deep and short triangular-shape on the left postorbital (Fig. 4c), consistent with adult *Albertosaurus* and *Gorgosaurus* specimens (Fig. 4e).

The shape of the frontal articular surface on the anterior ramus in TMP 2013.18.11 is different from that of TMP 1994.143.1. The anterior end of the frontal sutural surface in TMP 2013.18.11 is dorsoventrally expanded into a circular facet in posteromedial view (Fig. 2b,d), as in juvenile *Tyrannosaurus* (e.g., CMNH 7541 [Cleveland Museum of Natural History]). In large *Daspletosaurus*, this sutural facet is also circular in shape but is greater in depth. In contrast, the anterior end of the frontal sutural surface of TMP 1994.143.1 is dorsoventrally shallow and rectangular in posteromedial view (Fig. 3b,d), as in juvenile *Gorgosaurus* (TMP 1986.144.1). In larger *Gorgosaurus*, the anterior end of the frontal sutural surface is dorsoventrally expanded, albeit much less than in adult *Daspletosaurus*, and varies in shape from circular (e.g., ROM 1422 [Royal Ontario Museum]) to rectangular (e.g., TMP 1995.12.116).

The profile of the dorsal margin of the postorbital differs between TMP 2013.18.11 and TMP 1994.143.1. In TMP 2013.18.11, the dorsal margin of the postorbital is nearly straight above the anterior ramus and abruptly transitions to a convex region posterior to the approximate midpoint of the dorsal bar (Figs. 2 and 4a). In large specimens of *Daspletosaurus*, the entire profile of the dorsal margin is strongly convex with the apex located anterior to the midpoint of the dorsal bar (Fig. 4d). The degree of convexity in adult specimens is largely due to a swollen and extensive cornual process. In contrast, the shape of the dorsal profile of the postorbital in TMP 1994.143.1 and albertosaurines differs from those of *Daspletosaurus* in several aspects. In small juvenile *Gorgosaurus* and on the right postorbital of TMP 1994.143.1, the dorsal margin of the anterior ramus is distinctly concave, transitioning posteriorly to a convex region dorsal to the posterior ramus. As a result, the dorsal margin of the postorbital is distinctly sinusoidal. On the left postorbital of TMP 1994.143.1 and in large albertosaurines (Fig. 4c,e), the dorsal margin is only weakly sinusoidal, as the extremity of the anterior ramus is either gently concave (AMNH 5336) or flat (TMP 1995.12.115); the mid-region and posterior ramus are strongly convex, surpassing the height of the anterior ramus (Fig. 4c,e).

Some of the most obvious differences between the postorbital of TMP 2013.18.11 and TMP 1994.143.1 are in the cornual process. The cornual process in TMP 2013.18.11 is a raised C-shaped ridge that is separated from the posterodorsal corner of the orbit by a smooth surface of bone and occupies most of the lateral surface of the anterior ramus (Figs. 2a,c and 4a). Although the morphology of the cornual process in TMP 2013.18.11 is consistent with that of mature *Daspletosaurus* individuals, it differs in the following aspects: (1) it is less distinctive and covers a smaller area on the dorsal bar, (2) its surface is smooth and porous rather than rugose, (3) it does not extend above the dorsal surface of the postorbital, and (4) its posterior margin does not approach the laterotemporal fenestra (Fig. 2a,c). In contrast to TMP 2013.18.11 and *Daspletosaurus*, the cornual process of TMP 1994.143.1 and albertosaurines is located directly adjacent to the posterodorsal margin of the orbit and extends only a short distance onto the anterior ramus (Figs. 3 and 4b,c,e). In TMP 1994.143.1, the degree of development of the cornual process differs between the left and right postorbital: whereas it is a flat, rectangular, and textured region on the left element, it is expressed as a small, posteriorly-curling, semicircular lip on the right element. Despite these differences, the morphology of the cornual process is nearly identical to those observed in *Gorgosaurus*, where the left cornual process is more similar to that of juvenile individuals (Fig. 4a,b) and the right is similar to that of subadults and adults (Fig. 4c).

Aspects of the dorsal articular surface for the squamosal (DASS) of the postorbital differ between TMP 2013.18.11 and TMP 1994.143.1. In both TMP 2013.18.11 and mature *Daspletosaurus*, the DASS terminates posterior to the anterior margin of the laterotemporal fenestra and its anterior extremity smoothly transitions to the exposed dorsal margin of the postorbital, where it forms an uninterrupted and dorsally convex arc in lateral view^7,9^ (Figs. 2 and 4a,d). Furthermore, the DASS is dorsally or posterodorsally planar across its entire length, such that it is not visible in lateral view in these specimens. In contrast, the DASS of TMP 1994.143.1 extends anterior to the laterotemporal fenestra, as in albertosaurines, and is emarginated by a shallow notch at its anterior end. The depth of the notch in TMP 1994.143.1 (Fig. 3b) is intermediate between the condition found in juvenile *Gorgosaurus* (notch absent in TMP 1986.144.1: Fig. 4b) and adult *Gorgosaurus* individuals (notch deep in UALVP 10: Fig. 4e). In addition, the DASS of TMP 1994.143.1 and immature *Gorgosaurus* slopes ventrolaterally, such that it is visible in lateral view (Fig. 4b,c).

The morphology of the ventral ramus of the postorbital also differs between TMP 2013.18.11 and TMP 1994.143.1. In both TMP 2013.18.11 and mature *Daspletosaurus* specimens, the ventral ramus descends nearly vertically and is mostly straight, although it is narrower relative to the length of the dorsal bar anterior to the laterotemporal fenestra in TMP 2013.18.11 (Fig. 2). The width of the ventral ramus is slightly tapered between the dorsal bar and most posterior extent of the orbit in TMP 2013.18.11, but is of largely constant width in large *Daspletosaurus* specimens (Fig. 4d). Below the most posterior extent of the orbit, the ventral ramus of large *Daspletosaurus* bears a weakly-developed, obtuse-angled, anteriorly-projecting suborbital process that, unlike most other tyrannosaurids, does not inflect strongly into the orbit; as a result, the orbit in *Daspletosaurus* is oval in lateral view. Although the distal end of the ventral ramus is broken in TMP 2013.18.11, this feature tapers to an acute angle of less than or equal to 60° in adult *Daspletosaurus*. In contrast to the condition observed in TMP 2013.18.11 and *Daspletosaurus*, the ventral ramus of the postorbital in TMP 1994.143.1 and albertosaurines is wide just below the dorsal bar and tapers ventrally until it reaches the most posterior extent of the orbit, below which it retains a relatively constant width (Fig. 3). A subtle inflection on the posterior margin of the ventral ramus, located dorsal to the most posterior extent of the orbit, marks the dorsal end of the contact with the jugal (Figs. 3 and 4b,c,e). The distal extremity of the ventral ramus is rectangular in shape, giving rise to a prominent, right-angled, and blade-like suborbital process that expands anteriorly into the orbital fenestra (Fig. 4) and gives the orbit a distinctive “comma” shape. The ventral ramus is strongly curved between the anterior extremity of the dorsal bar and the suborbital process, such that the orbital margin between these features is nearly semicircular.

Finally, although the distal end of the posterior ramus is missing in TMP 2013.18.11, this feature is seen to differ significantly between TMP 1994.143.1 and other *Daspletosaurus* specimens. Whereas the posterior ramus is long and terminates at the level of or posterior to the posterodorsal margin of the laterotemporal fenestra in *Daspletosaurus*, it is short and terminates anterior to the posterodorsal margin of the laterotemporal fenestra in TMP 1994.143.1 (Fig. S3), which is a synapomorphy of Albertosaurinae.

### Preservation of TMP 1994.143.1

Although it is well preserved, the skull of TMP 1994.143.1 has undergone burial deformation that altered certain morphological aspects. The articulated rostrum, consisting of the premaxillae, maxillae, nasal, right jugal, and right lacrimal, was preserved upside down, whereas all other skull bones were found disarticulated. As a consequence, the rostrum is dorsoventrally compressed and splayed laterally (Fig. 1c,d), giving the skull a wider temporal region than is typical for albertosaurines^4,20^ and distorting some elements/features (lacrimal, maxillary fenestra; Supplementary Information). As such, the anatomical features of TMP 1994.143.1 need to be carefully examined in order to determine the appropriate character state codings for phylogenetic analyses.

### Phylogenetic analyses

**Figure 5:**
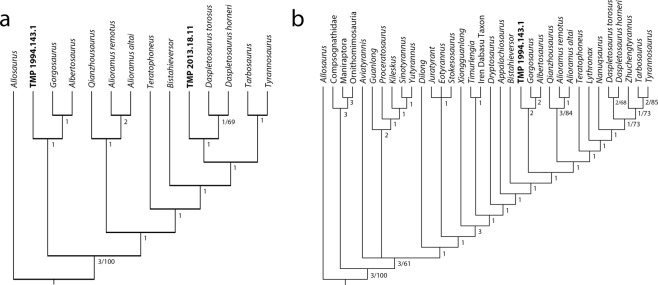
Consensus of the cladistic analyses of Tyrannosauroidea, based on the matrix of Carr *et al*. (2017). (**a**) Single most parsimonious tree recovered from an analysis of characters pertaining only to the postorbital, which evaluates the positions of TMP 2013.18.11 and TMP 1994.143.1. Tree length = 18 steps, consistency index = 0.778, retention index = 0.949. (**b**) Single most parsimonious tree recovered from an analysis including cranial and postcranial characters, which evaluates the position of TMP 1994.143.1. Tree length = 833 steps, consistency index = 0.539, retention index = 0.796. Numbers adjacent to nodes constitute Bremer support values (<3) and Bootstrap values (>50). Unsupported nodes are collapsed. Institutional abbreviations: TMP, Royal Tyrrell Museum of Vertebrate Palaeontology.

The morphological differences observed between the new juvenile tyrannosaurid postorbital (TMP 2013.18.11) and the postorbital of the purported juvenile *Daspletosaurus* skull (TMP 1994.143.1), as well as the morphological similarities observed between TMP 1994.143.1 and *Gorgosaurus* specimens encourage a reassessment of specimen identifications. Phylogenetic analyses were conducted to assess the taxonomic affinities of these two specimens using the character matrix for Tyrannosauroidea of Carr and colleagues^9^ (see Supplementary Tables S2–S4). First, a parsimony analysis of characters pertaining only to the postorbital element of tyrannosaurids produced a single most parsimonious tree of 18 steps, which reveals that TMP 2013.18.11 is a sister taxon to *Daspletosaurus* spp., whereas TMP 1994.143.1 is retrieved as a member of Albertosaurinae (Fig. 5a). Following this result, the entire skull of TMP 1994.143.1 was coded for a parsimony analysis of tyrannosauroids, which produced a single most parsimonious tree of 833 steps that again placed TMP 1994.143.1 within the Albertosaurinae clade as sister to *Albertosaurus* and *Gorgosaurus* (Fig. 5b). In this second analysis, an additional 48 steps is required to move TMP 1994.143.1 to a position as sister taxon to *Daspletosaurus* spp. These phylogenetic analyses reveal that TMP 1994.143.1, even though it is a juvenile individual, possesses most of the previously recognized synapomorphies of Albertosaurinae, including both autapomorphies of *Gorgosaurus libratus*^9^ (see Supplementary Table S1). On the contrary, the specimen possesses few tyrannosaurine or *Daspletosaurus* synapomorphies, all of which can be attributed to either taphonomic deformation, ontogenetically variable characters, or individual variation (Supplementary Information).

## Discussion

**Figure 6:**
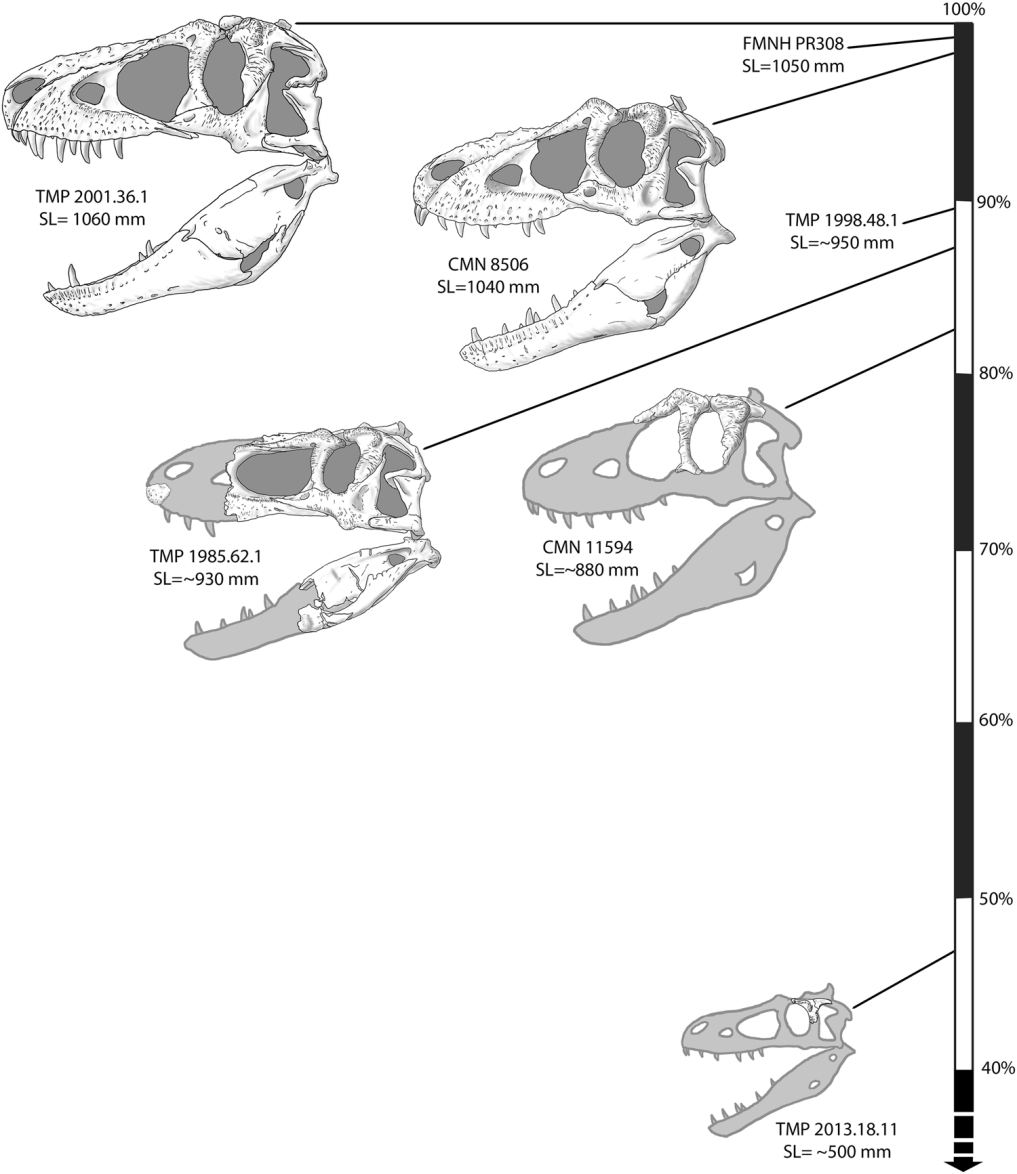
Ontogenetic series of *Daspletosaurus torosus* including TMP 2013.18.11 and other key specimens. Specimens are plotted based on percent skull length (SL) relative to the largest known individual (TMP 2001.36.1). Specimens are drawn to scale. Some specimens are listed but not illustrated due to space limitations. Note that this taxon is predominantly known from large individuals (SL > 80%). Institutional abbreviations: CMN, Canadian Museum of Nature; FMNH, Field Museum of Natural History; TMP, Royal Tyrrell Museum of Palaeontology.

The discovery and description of a small tyrannosaurid postorbital, TMP 2013.18.11, sheds light on the taxonomic affinity of TMP 1994.143.1, a specimen heretofore regarded as the only known juvenile *Daspletosaurus* skeleton. The new postorbital possesses several features that clearly differentiate it from the postorbital of TMP 1994.143.1 and *Gorgosaurus* but link it with *Daspletosaurus*. Anatomical comparisons and phylogenetic analyses reveal that TMP 2013.18.11 can be confidently assigned to a juvenile *Daspletosaurus*, possibly *D*. *torosus*, whereas TMP 1994.143.1 is attributable to the sympatric albertosaurine *Gorgosaurus libratus*. This re-identification results in removal of the only known juvenile skull from the *Daspletosaurus* ontogenetic series, leaving only those of larger individuals (>80% maximum skull length; Fig. 6).

The isolated postorbital TMP 2013.18.11 represents only the second known juvenile element of *Daspletosaurus* in North America (a small dentary from a similar-sized *D*. *horneri* individual is known from Montana, see Carr and colleagues^9^). The size of the element is similar to that of the smallest known *Gorgosaurus* skulls (~500 mm in length), but is considerably more robust. Furthermore, the cornual process is more developed and is both absolutely and relatively larger than that of similar-sized juvenile *Gorgosaurus* (Fig. 4) or even that of larger individuals of *Albertosaurus* (TMP 2008.75.53), *Alioramus*^21,22^ (MPC 100/1844), *Gorgosaurus*^4^ (TMP 1991.36.500), and *Tyrannosaurus rex*^2,23^ (CMNH 7541). The presence of a pronounced cornual process in small juvenile *Daspletosaurus* individuals is consistent with the fact that this taxon possesses the largest and most extensive cornual process of all tyrannosaurids^9^, and suggests that the growth of this feature either began at an earlier age or occurred at a faster rate than in other tyrannosaurids.

The new postorbital, TMP 2013.18.11, provides insights into early *Daspletosaurus* ontogeny. As in other tyrannosaurids^2,4,8^, the postorbital of *Daspletosaurus* changes significantly through growth, most dramatically in the increase in size and rugosity of the cornual process. In small juvenile *Daspletosaurus* individuals (TMP 2013.18.11), the cornual process is restricted to a relatively smooth ridge along the anterior mid-region of the dorsal bar, whereas it expands into a highly rugose and inflated boss that covers most of the dorsal bar in larger individuals^4,9,19^ (Figs. 3b and 4a,d). Through ontogeny, the postorbital also undergoes a significant increase in the depth of the anterior ramus and the frontal sutural surface, as well as increases in the posterodorsal slope of the frontal sutural surface, in the convexity of the dorsal margin (primarily above the anterior ramus), and in the width of the ventral ramus (Fig. 4a,d). Although the postorbital undergoes similar ontogenetic changes in albertosaurines^2,6^ and the tyrannosauroid *Bistahieversor*^7,24^, the changes observed in *Daspletosaurus* are extreme and are most comparable to those of other large tyrannosaurines (e.g., *Tarbosaurus*^8^, *Tyrannosaurus*^2,6^).

Finally, our study reveals that previously unrecognized morphological differences exist between juvenile albertosaurines and tyrannosaurines and demonstrates that juvenile tyrannosaurids are more morphologically distinct than originally thought. Previous issues associated with differentiating juveniles of these two clades were likely caused by the misidentification of TMP 1994.143.1 as a juvenile *Daspletosaurus*^4^. Despite general similarities in cranial features^2,4,8,9,17,19,25^, here we show that characteristics of the postorbital alone are sufficient to determine the taxonomic identity of juvenile tyrannosaurids; we also hypothesize that other elements may be equally diagnostic. Additionally, the taxonomic affinity of juvenile-subadult individuals can be determined using phylogenetic analyses, although less mature tyrannosaurids (<50% skull length of largest individual) can erroneously be recovered as basal tyrannosauroids due to the delayed ontogenetic development of several derived characters^8^. For such young individuals, it may be best to rely on the presence of apomorphies or other diagnostic features to determine taxonomic affinity.

## Conclusions

The discovery of an isolated juvenile tyrannosaurid postorbital prompted a reassessment of a specimen previously identified as a juvenile *Daspletosaurus*, TMP 1994.143.1. Based on phylogenetic analyses, the presence of several albertosaurine features and the absence of tyrannosaurine features, TMP 1994.143.1 is re-identified as a juvenile-subadult specimen of *Gorgosaurus libratus*. The taxonomic reassignment of TMP 1994.143.1 alters our understanding of *Daspletosaurus* ontogeny and may require a re-evaluation of studies that considered the specimen a juvenile *Daspletosaurus*. The newly identified juvenile postorbital, TMP 2013.18.11, reveals that *Daspletosaurus* postorbital ontogeny is similar to that of other large tyrannosaurines, although the cornual process appears to develop earlier or faster in this taxon than in other tyrannosaurids. Our findings reveal that many tyrannosaurid apomorphies are present in ontogenetically young individuals and that juvenile forms of tyrannosaurine and albertosaurine species are easier to distinguish from one another than previously thought.

## Materials and Methods

Tyrannosaurid specimens from upper Campanian and Maastrichtian deposits of Alberta and Montana housed at various institutions were examined and compared (see institutional abbreviations below). Measurements were taken using digital calipers and measuring tape. Material examined included juvenile to adult specimens of the taxa *Albertosaurus sarcophagus*, *Daspletosaurus torosus*, *D*. *horneri*, *Gorgosaurus libratus*, and *Tyrannosaurus rex*. Comparative work primarily focused on the postorbital, which is a useful element for determining taxonomic identity as it possesses a suite of morphological features diagnostic to the genus and, in some cases, species level (*sensu* Carr and colleagues^9^; JTV, pers. obs.). Digital renderings of TMP 1994.143.1 skull elements were produced in Amira v.5.3.3 for visualization, articulation of elements, and in some cases, character scoring (Supplementary Information).

### Phylogenetic analysis and apomorphy comparison

TMP 1994.143.1 and TMP 2013.18.11 were scored in the character dataset of Carr and colleagues^9^ using Mesquite v.3.2 and exported into TNT v.1.5. For this study, we used parsimony analysis using new technology search with sectorial search, ratchet, tree drift, and tree fusing all at default settings. The analysis was set to recover most parsimonious trees in 10 replicates. Trees were rooted with *Allosaurus* as the outgroup. Tree support was calculated with standard bootstrap resampling (1000 replicates) and Bremer supports. A first phylogenetic analysis tested the relationship of tyrannosaurids, *Bistahieversor*, TMP 1994.143.1, and TMP 2013.18.11 based on characters pertaining to the postorbital exclusively (Carr *et al*.^9^: characters 110–120). This analysis recovered only one most parsimonious tree; TBR branch swapping recovered no additional trees. A second phylogenetic analysis sought to further test the relationship of TMP 1994.143.1 with other tyrannosauroids and included all tyrannosauroid taxa (except ‘*Raptorex’*) and all characters from the Carr *et al*.^9^ dataset. One most parsimonious tree was recovered from the new technology search; TBR branch swapping recovered no additional trees.

Character scorings of TMP 1994.143.1 were compared with various apomorphies recognized for tyrannosaurid clades and taxa (sensu Carr and colleagues^9^). Specifically, the focus was on 11 synapomorphies of the genus *Daspletosaurus*, five autapomorphies of *Daspletosaurus torosus*, nine synapomorphies of Albertosaurinae, two autapomorphies of *Gorgosaurus libratus*, and several synapomorphies for more inclusive clades within Tyrannosaurinae (Supplementary Table S1).

## Supplementary information

Supplementary Information

Supplementary Character Dataset

## Data Availability

Supporting data, including character scorings of TMP 2013.18.11 and TMP 1994.143.1, are included in the supplementary materials. Character descriptions as in Carr and colleagues^9^. Locality and specimen information for TMP 2013.18.11, TMP 1994.143.1 are on file in collections at the Royal Tyrrell Museum of Palaeontology.
