# Supplementary material for: Reassessment of a juvenile Daspletosaurus from the Late Cretaceous of Alberta, Canada with implications for the identification of immature tyrannosaurids
Source: Sci Rep. 2019 Nov 28;9:17801. doi: 10.1038/s41598-019-53591-7 (PMC6882908; doi:10.1038/s41598-019-53591-7)
Supplement: Supplementary file 1 — Supplementary Information [file 41598_2019_53591_MOESM1_ESM.docx]

Supplementary Information for:

**Reassessment of a juvenile *Daspletosaurus* from the Late Cretaceous of Alberta, Canada with implications for the identification of immature tyrannosaurids**

Jared T. Voris^1*^, Darla K. Zelenitsky^1^, François Therrien^2^, Philip J. Currie^3^

^1^University of Calgary, Department of Geoscience, Calgary, Alberta, Canada T2N 1N4

^2^Royal Tyrrell Museum of Palaeontology, Drumheller, Alberta, Canada T0J 0Y0

^3^University of Alberta, Biological Sciences, Edmonton, Alberta, Canada T6G 2R3

**Synapomorphies and other features of TMP 1994.143.1**

Evaluation of the presence/absence of diagnostic tyrannosaurid cranial characters identified by Carr and colleagues^1^ in TMP 1994.143.1 reveals that this specimen is more similar to both juvenile and adult specimens of *Gorgosaurus* *libratus* than to any specimen of *Daspletosaurus* (Table S1). Of the eight recognized Albertosaurinae synapomorphies that can be assessed in TMP 1994.143.1 (Figs. S1-S6), five definitively exhibit the albertosaurine condition and three are ambiguous due to their ontogenetic variability between juvenile and adult *Gorgosaurus* specimens. These ambiguous characters include the ventral contact between the jugal and quadratojugal (Carr et al^1^: character 106), the depth of the notch at the anterior tip of the DASS (Carr et al^1^: character 114), and the inclination of the vomeropterygoid neck (Carr et al^1^: character 180). First, the ventral contact between the jugal and quadratojugal in TMP 1994.143.1 possesses a shallow anterodorsal inflection (Fig. S2), a condition intermediate to the steep anterodorsally inclined (~45°) inflection diagnostic of adult albertosaurines and the horizontal (anteroposterior) contact of small juvenile albertosaurines (e.g., TMP 2016.14.1) and of tyrannosaurines. Secondly, TMP 1994.143.1 possesses a shallow notch at the anterior tip of the DASS (dorsal articular surface of squamosal; Figs. 3, S3) rather than the deep notch diagnostic of albertosaurines. However, this character is observed to be ontogenetically variable among albertosaurines: whereas small juveniles (e.g. TMP 1986.144.1, TMP 2009.12.14) lack any significant concavity, larger juvenile individuals (e.g. TMP 1991.36.500 and TMP 1994.143.1) possess a shallow notch, and the largest individuals possess the diagnostic deep notch (e.g. TMP 1981.10.1, TMP 1995.12.116). Finally, TMP 1994.143.1 displays both the albertosaurine and tyrannosaurine condition with respect to the inclination of the vomeropterygoid neck, where the left palatine displays the tyrannosaurine condition (anterodorsal inclination) and the right palatine displays the albertosaurine condition (dorsal inclination) (Fig. S5). The presence of both conditions may reflect the transitional ontogenetic stage of TMP 1994.143.1, intermediate between small juvenile *Gorgosaurus* specimens that exhibit the tyrannosaurine condition in both palatines (e.g., TMP 2009.12.14, TMP 2016.14.1), and larger individuals that display the albertosaurine condition (e.g. TMP 1991.36.500). Given that these three albertosaurine synapomorphies are shown here to be acquired gradually during ontogeny, their absence in TMP 1994.143.1 does not reflect a tyrannosaurine affinity for the specimen but rather its juvenile ontogenetic status.

When TMP 1994.143.1 is examined for the presence of recognized synapomorphies of the genus *Daspletosaurus* (Carr et al^1^: sup. info), it appears to possess two of the 10 synapomorphies assessable in the specimen (Table 2.2; Figs. 3, S5, S6-S12). However, closer examination reveals that one of these two features does not exhibit the typical morphology observed in *Daspletosaurus* specimens and the other feature is not unique to *Daspletosaurus* but is also present in immature *Gorgosaurus* specimens. First, although TMP 1994.143.1 possesses a distinct ridge dorsal to the epipterygoid fossa on the laterosphenoid, a *Daspletosaurus* synapomorphy recognized by Carr et al^1^ (character 219), this ridge is short anteroposteriorly and unpronounced in comparison to all other *Daspletosaurus* specimens (Fig. S10). Due to these differences, this feature is regarded as an individual variation in TMP 1994.143.1 rather than homologous with the *Daspletosaurus* synapomorphy. Secondly, whereas the primary palatine pneumatopore is located posterior to the anterior margin of the vomeropterygoid neck in TMP 1994.143.1, as in *Daspletosaurus* (Carr et al^1^: character 184), this is also the case in juvenile *Gorgosaurus* (e.g. TMP 1986.144.1, TMP 2009.12.14; Fig. S5). In addition, the primary palatine pneumatopore is located much more posteriorly in *Daspletosaurus* than in either TMP 1994.143.1 or juvenile *Gorgosaurus*. As a result, the posterior position of the primary palatine pneumatopore in TMP 1994.143.1 is regarded as ontogenetic variation rather than a taxonomic indicator.

Additional examination of the synapomorphies of several tyrannosaurine clades further support the non-tyrannosaurine affinity of TMP 1994.143.1 (Table S1, Figs. S2, S3, S5, S7, S9, S10, S12-S15). The specimen exhibits only four of the 26 synapomorphies of the tyrannosaurine clades that include *Daspletosaurus*, three of which are likely due to either taphonomic deformation (n=1) or individual variation (n=2), whereas a fourth may be pathological or individual variation. First, the subnarial process of TMP 1994.143.1 is horizontal and mostly concealed in lateral view (Fig. 1), which would be considered a tyrannosaurine synapomophy (Carr et al^1^: character 14), but the condition in TMP 1994.143.1 is likely due to the fact that the rostrum of the specimen is dorsoventrally compressed due to burial deformation. Second, although the ridge delimiting the lateral margin of the supratemporal fossa on the dorsal surface of the squamosal is partially divided by a median groove in TMP 1994.143.1 (Fig. S13), a condition that approaches the complete division that is a tyrannosaurine synapomorphy (Carr et al^1^: character 122), it is only present on the right element and a groove is absent in the left element. Because division of the ridge is only partial and occurs only on the right side, it likely represents individual variation in TMP 1994.143.1 rather than the tyrannosaurine condition. Thirdly, the ectopterygoid pneumatopore is circular in the left ectopterygoid (Fig. S14), which is a synapomorphy of Tyrannosaurinae (Carr et al^1^: character 177), but the right ectopterygoid possesses an oval-shaped pneumatopore. The presence of a circular ectopterygoid pneumatopore in an immature *Gorgosaurus* individual (TMP 1991.36.500) suggests this feature may be individually variable in albertosaurines. Finally, although the sagittal crest on the frontal is slightly taller in TMP 1994.143.1 than typically seen in albertosaurines (Fig. S15), which is a tyrannosaurine characteristic (Carr et al^1^: character 157), it is similar in proportions to that of another smaller, immature *Gorgosaurus* (TMP 2009.12.14) and is even relatively shorter than that of the adult *Gorgosaurus* UALVP 10 (Fig. S14). Consequently, the prominence of the crest in TMP 1994.143. may simply be due to individual variation or, alternatively, taphonomic deformation as both frontals are laterally compressed.

**Table S1.** Apomorphies for Albertosaurinae, *Gorgosaurus*, *Daspletosaurus*, and various clades within Tyrannosaurinae, in comparison with the character state for TMP 1994.143.1. “Juvenile *Gorgosaurus*” refers to the following specimens: TMP 1986.144.1, TMP 1991.36.500, TMP 2009.12.14, and TMP 2016.14.1.

|  | Character | *Daspletosaurus* | *Gorgosaurus* | Juvenile  *Gorgosaurus* | TMP 1994.143.1 | Associated Figure |
| --- | --- | --- | --- | --- | --- | --- |
| Albertosaurinae | **Lacrimal**, Form of the joint surface for the frontal in medial view (Character 86) | Medially-deflected conical projection | Dorsoventrally flat | Dorsoventrally flat | Dorsoventrally flat | Fig. S1 |
|  | **Jugal**, Angle of the posterior margin of the suture with lacrimal in lateral view (Character 96) | Shallow in *D. torosus*; Steep in *D. horneri* | Steep | Steep | Steep | Fig. S2 |
|  | **Jugal**, Slope of the ventral margin of quadratojugal joint surface angle in lateral view (Character 106) | Approximately anteroposterior; nearly straight | Prominent anterodorsal inflection of approximately 45° | Nearly straight (TMP 1986.144.1) or shallow anterodorsal inflection (TMP 2009.12.14) | Shallow anterodorsal inflection | Fig. S2 |
|  | **Postorbital**, form of the posterodorsal margin of the squamosal ramus in lateral view (Character 114) | Shallow concave notch or uninterrupted convex arc | Emarginated by squamosal (discrete notch within margin) | shallow concave notch | Shallow to deep concave notch | Fig. 2, S3 |
|  | **Postorbital**, Extent of the squamosal ramus relative to posterodorsal margin of lateral temporal fenestra (Character 115) | Reaches or extends posterior to margin | Terminates anterior to margin | Terminates anterior to margin | Terminates anterior to margin | Fig. S3 |
|  | **Quadrate**, Orientation of the medial margin of the quadratojugal articular surface where the quadratojugal wraps around the quadrate lateral condyle (Character 147) | vertical or dorsomedial | dorsolateral | dorsolateral | dorsolateral | Fig. S4 |
|  | **Palatine**, Orientation of the neck of the vomeropterygoid process in lateral view (Character 180) | inclined anterodorsally | Vertical | inclined anterodorsally in some specimens (e.g. TMP 2009.12.14 and TMP 2016.14.1); vertical in others (e.g. TMP 1986.144.1) | left element is inclined anterodorsally; right element is approximately vertical | Fig. S5 |
|  | **Basisphenoid**, Inflation of the ceiling of the basisphenoid recess in ventral view (Character 210) | Present | Absent | Absent | Absent | Fig. S6 |
| *Gorgosaurus libratus* | **Frontal**, Shape of the frontal in mediolateral cross section ahead of dorsotemporal fossa | Slope towards interfrontal suture; concave | Slope away from interfrontal suture; convex | slope away from interfrontal suture; convex | slope away from interfrontal suture; convex | Fig. S7 |
|  | **Jugal**, Anteroposterior length of the basal postorbital process relative to the dorsoventral depth of the suborbital region | Postorbital process anteroposteriorly longer than suborbital depth | Postorbital process shorter than suborbital depth | Postorbital process shorter than suborbital depth | Postorbital process length approximately equal to suborbital depth | Fig. S2 |
| *Daspletosaurus* | **Maxilla**, Form of the subcutaneous surface texture in lateral view (character 43). | Extremely coarse; deep sulci anterior of antorbital fossa | Random foramina and shallow grooves and ridges | Random foramina and either smooth (TMP 2016.14.1) or random foramina and shallow grooves and ridges (e.g. TMP 1991.36.500) | Random foramina and shallow grooves and ridges | Fig. S8 |
|  | **Lacrimal**, Presence of an accessory posteriorly extending cornual process on the lateral surface between the cornual process and the supraorbital ramus (Character 72) | present | absent | absent | absent | Fig. S9 |
|  | **Lacrimal**, Exposure of the maxillary process of anterior ramus in lateral view (Character 89). | Only ventral margin visible | both dorsal and ventral margins visible | both dorsal and ventral margins visible | both dorsal and ventral margins visible | Fig. S9 |
|  | **Postorbital**, cornual process, position relative to laterotemporal fenestra, lateral view (Character 113) | Approaches fenestra | Does not approach fenestra | Does not approach fenestra | Does not approach fenestra | Fig. 2 |
|  | **Squamosal**, Position of the anterior tip of the dorsal postorbital process (Character 130) | Posterior to anterior margin of lateral temporal fenestra | Level with or anterior to anterior margin of laterotemporal fenestra | Level with or anterior to anterior margin of laterotemporal fenestra | Anterior to anterior margin of laterotemporal fenestra | Fig. S3 |
|  | **Frontal**, nasal process, ridge that extends anteroposteriorly along the process, presence, dorsal view (Character 154). | Present | absent | absent | absent | Fig. S7 |
|  | **Vomer**, Presence of deep keel below stem | Present | Present in some specimens (e.g. TMP 2000.13.11) | present in some specimens (TMP 2009.12.14) | ? | NA |
|  | **Palatine**, primary opening of palatine recess, location of anterior margin, lateral view (Character 184). | Level with or posterior to recess | Anterior to recess | Posterior to recess | Posterior to recess | Fig. S5 |
|  | **Laterosphenoid**, mediolaterally oriented ridge on dorsolateral surface that extends laterally to the edge of the dorsotemporal fenestra (Character 219). | Present; extends onto capitate process | Absent | Absent | Present; does not extend onto capitate process | Fig. S10 |
|  | **Dentary**, position of the transition point between the ventral and anterior margins of the bone, lateral view (Character 229). | Below alveoli 1-3 | Below the fourth alveolus | Below the fourth alveolus | Below the fourth alveolus | Fig. S11 |
|  | **Maxillary teeth**, number of teeth (Character 261). | 15-17 | 13-15 | 13-14 | 13 | Fig. S12 |
| *D. torosus* | **Lacrimal**, anterior ramus, length relative to the ventral ramus (Character 74) | Longer than ventral ramus; terminates near anterior margin of antorbital fenestra | shorter than ventral ramus | longer than the ventral ramus | Approximately equal to ventral ramus; terminates far posterior to anterior margin of ant. fen. | Not labeled; visible in Fig. S12 |
|  | **Lacrimal**, transition between antorbital fossa and the subcutaneous surface of the ventral ramus, form, lateral view (Character 77). | Variable: fossa is continuous (e.g. TMP 2001.36.1) or deeply inset (specimen #?) | Fossa is deeply inset forming a ridge along subcutaneous surface | Fossa is deeply inset forming a ridge along subcutaneous surface | Fossa is deeply inset forming a ridge along subcutaneous surface | Fig. S11 |
|  | **Surangular**, adductor muscle attachment site dorsal to surangular shelf, orientation, lateral and dorsal views (Character 244) | Faces almost equally dorsally and laterally | Faces almost equally dorsally and laterally | Faces almost equally dorsally and laterally | Faces almost equally dorsally and laterally | Not figured |
| Tyrannosaurinae | **Premaxilla**, Orientation of the subnarial (maxillary) processes of the premaxilla (Character 14) | Dorsal; processes mostly obscured in lateral view | Dorsolateral | Dorsolateral; most juvenile skulls are laterally compressed | Dorsal-dorsolateral; specimen is dorsoventrally compressed however | Not labeled; visible in Fig. S12 |
|  | **Maxilla**, Depth of the joint surface for the palatine (Character 40) | Deep; conceals tooth root bulges in medial view | Shallow; tooth root bulges visible in medial view | Shallow; tooth root bulges visible in medial view | Shallow, tooth root bulges visible in medial view | Not figured |
|  | **Nasal**, extent of premaxillary process apposition (Character 54) | Separated for most of their length | Apposed for most of their length; abruptly separate at tip | Apposed for most of their length; abruptly separate at tip | Apposed for most of their length; abruptly separate at tip | Unmarked; Fig. 1 |
|  | **Lacrimal**, extent of anterodorsal process (Character 81) | Long | Generally short | generally short | short on left; long on right (length comparable to *Gorgosaurus* TMP 1994.12.602) | Not labeled; Fig. 1, S9, S12 |
|  | **Squamosal**, morphology of the surface of the dorsotemporal fossa (Character 123) | Convex | Flat or concave | flat | flat | Not labeled Fig. S13 |
|  | **Squamosal**, morphology of the anterior tip of the quadratojugal process (Character 125) | Squared off | Tapered | Tapered | Tapered | Not labeled; Fig. S3 |
|  | **Squamosal**, presence of a pneumatic recess in ventral view (Character 131) | Recess present; edges are undercut | Fossa instead of recess; edges are not undercut | Fossa instead of recess; edges are not undercut | Fossa instead of recess; edges are not undercut | Not figured |
|  | **Ectopterygoid**, inflation of the ectopterygoid body and pterygoid process (Character 174) | Inflated | Uninflated | Uninflated | Uninflated | Not figured |
|  | **Ectopterygoid**, shape of the pneumatopore in ventral view (Character 177) | Large and round or triangular | Thin and narrow slit | Thin and narrow slit | Thin and narrow slit | Fig. S14 |
|  | **Ectopterygoid**, surface morphology of floor immediately adjacent to pneumatopore (Character 178) | Pneumatopore and floor separated by a shelf | Flat | Flat | Flat | Not labeled; Fig. S14 |
|  | **Palatine**, presence of brace in the anteroventral corner of the jugal articulation (Character 188) | Present | Absent; maxilla contacts anteroventral corner of jugal contact | Absent; maxilla contacts anteroventral corner of jugal contact | Absent; maxilla contacts anteroventral corner of jugal contact | Not labeled; Fig. S5 |
|  | **Laterosphenoid**, morphology of the antotic crest, anterior to the epipterygoid fossa in anterior and lateral views (Character 216) | Robust and rugose | Indistinct | Indistinct | Indistinct | Fig. S10 |
| Derived Tyrannosaurines | **Postorbital**, position of the cornual process relative to the orbital margin (Character 112) | Posterodorsal; Separated from orbital margin by smooth region of bone | Adjacent to orbit | Adjacent to orbit | Adjacent to orbit | Fig. 3 |
|  | **Squamosal**, morphology of the ridge delimiting the lateral margin of the dorsotemporal fossa (Character 122) | Divided into pair of ridges | Undivided | Undivided | Left is undivided; right is partially divided by a short and shallow groove | Fig. S13 |
|  | **Quadratojugal**, extent of the dorsal quadratojugal process of jugal relative to base of the quadratojugal stem in the posteroventral corner of the laterotemporal fenestra (Character 134) | Approaches quadratojugal stem | Does not approach quadratojugal stem | Does not approach quadratojugal stem | Does not approach quadratojugal stem | Not figured |
|  | **Quadratojugal**, shape of the anterior tip of the jugal process (Character 135) | Squared off | Rounded | Tapered | Rounded | Not figured; rounded articular surface can be seen on jugal in Fig. S2 |
|  | **Frontal**, morphology of the sagittal crest (Character 135) | Dorsoventrally tall and unpaired | present as a slight midline bulge; variable | present as a slight midline bulge; variable; moderately tall in TMP 2009.12.14 | Moderately tall | Fig. S15 |
|  | **Frontal**, anteroposterior length of the sagittal crest relative to the entire length of the frontal (Character 158) | >25% | <15% | Much less than 15% | <15% | Not labeled; visible in Fig. S7 |
|  | **Dentary**, Angle between the anterior margin versus the dorsal margin (Character 237) | Approximately 90° | Approximately 45° or less | Less than 45° | Approximately 45° | Not labeled; visible in Fig. S11 |
|  | **Surangular**, morphology of the surangular shelf over the posteroventral foramen (Character 241) | Shelf projects ventrolaterally and overhangs foramen | Dorsal margin of foramen is in contact with shelf but shelf projects laterally and does not overhang | Dorsal margin of foramen is in contact with shelf but shelf projects laterally and does not overhang | Dorsal margin of foramen is in contact with shelf but shelf projects laterally and does not overhang | Not figured |
|  | **Angular**, morphology of the ventral margin (Character 248) | Anterior region flexed at posterior dentary contact such that there is distinct angle | Smoothly convex | Smoothly convex | Smoothly convex | Not figured |
| *DaspletosauRus*+Tyrannosaurini | **Maxilla**, exposure of the promaxillary fenestra in lateral view (Character 20) | Concealed by lateral lamina in lateral view | Visible | Visible | Concealed due to crushing but likely visible due to position of max. fen. | Unmarked; visible in Fig. 1, S12 |
|  | **Maxilla**, rotroposterior length of maxillary fenestra relative to the maximum length of the antorbital fossa ahead of the anterior-most point of the antorbital fenestra (Character 24) | Greater than 50% and also greater than 50% the length of the eyeball-bearing portion of the orbit | Less than 50% | Less than 50% | Less than 50% | Not labeled; visible in Fig. 1, S12 |
|  | **Maxilla**, morphology of nasal contact in subadult-adult specimens* (Character 42) | Deeply scalloped with several interlocking tongue-and-groove joints | Weakly scalloped | Weakly scalloped | Weakly scalloped (where visible); no interlocking tongue-and-groove joints | Not labeled; visible in Fig. S7, S12 |
|  | **Nasal**, constriction of frontal ramus (Character 59) | Constricted to less than half the width of the widest point of the nasal | Unconstricted | Unconstricted | Unconstricted; greater than 50% maximum width of nasal; nasal dorsoventrally compressed and laterally flared | Not labeled; Fig. 1 |
|  | **Jugal**, posterior extent of the antorbital fossa on the maxillary ramus (Character 91) | Does not extend past the jugal pneumatopore | Undercuts pneumatopore and extends behind pneumatopore, posterodorsal margin may be resorbed | Undercuts pneumatopore and extends behind pneumatopore to tip of anterior ascending process | Undercuts pneumatopore and extends behind pneumatopore to tip of anterior ascending process | Not labeled; Fig. S2 |
|  | **Jugal**, orientation of the long axis of the jugal pneumatopore (Character 93) | Nearly horizontal | Anteroposteriorly inclined at nearly 45° from horizontal | Anteroposteriorly inclined at nearly 45° from horizontal | Anteroposteriorly inclined at nearly 45° from horizontal | Not labeled; partially visible in Fig. S2 |
|  | **Jugal**, morphology of the fossa located at the base of the postorbital ramus (Character 97) | Deep | Shallow | flat; fossa absent or undeveloped | flat | Not labeled; Fig. S2 |

**Supplementary Figures**

**
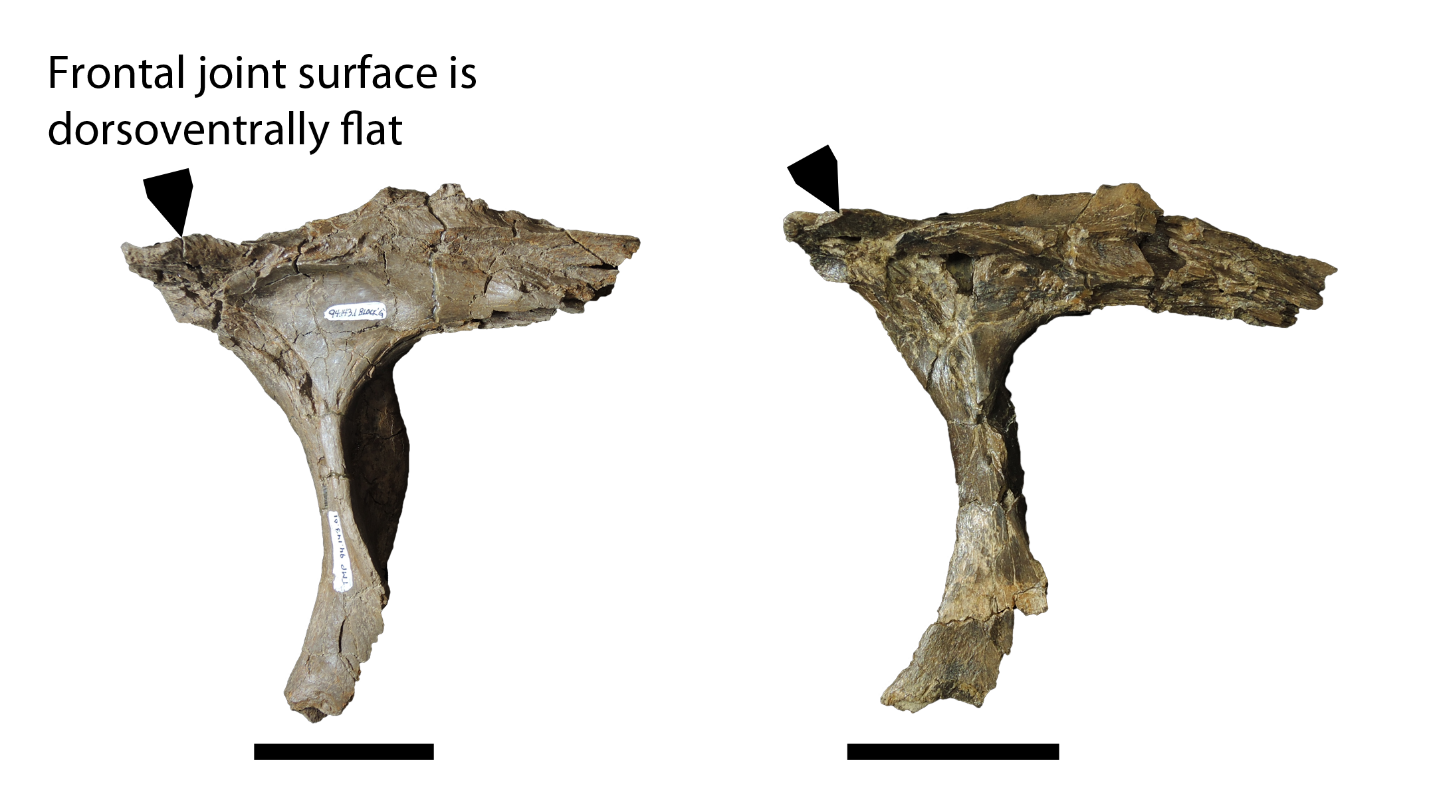
**

**Figure S1.** Left lacrimal of TMP 1994.143.1 (left) and TMP 1986.144.1 (right) in medial view. In *Daspletosaurus*, the frontal joint surface is conical. Scale bar equals 50 mm.

**
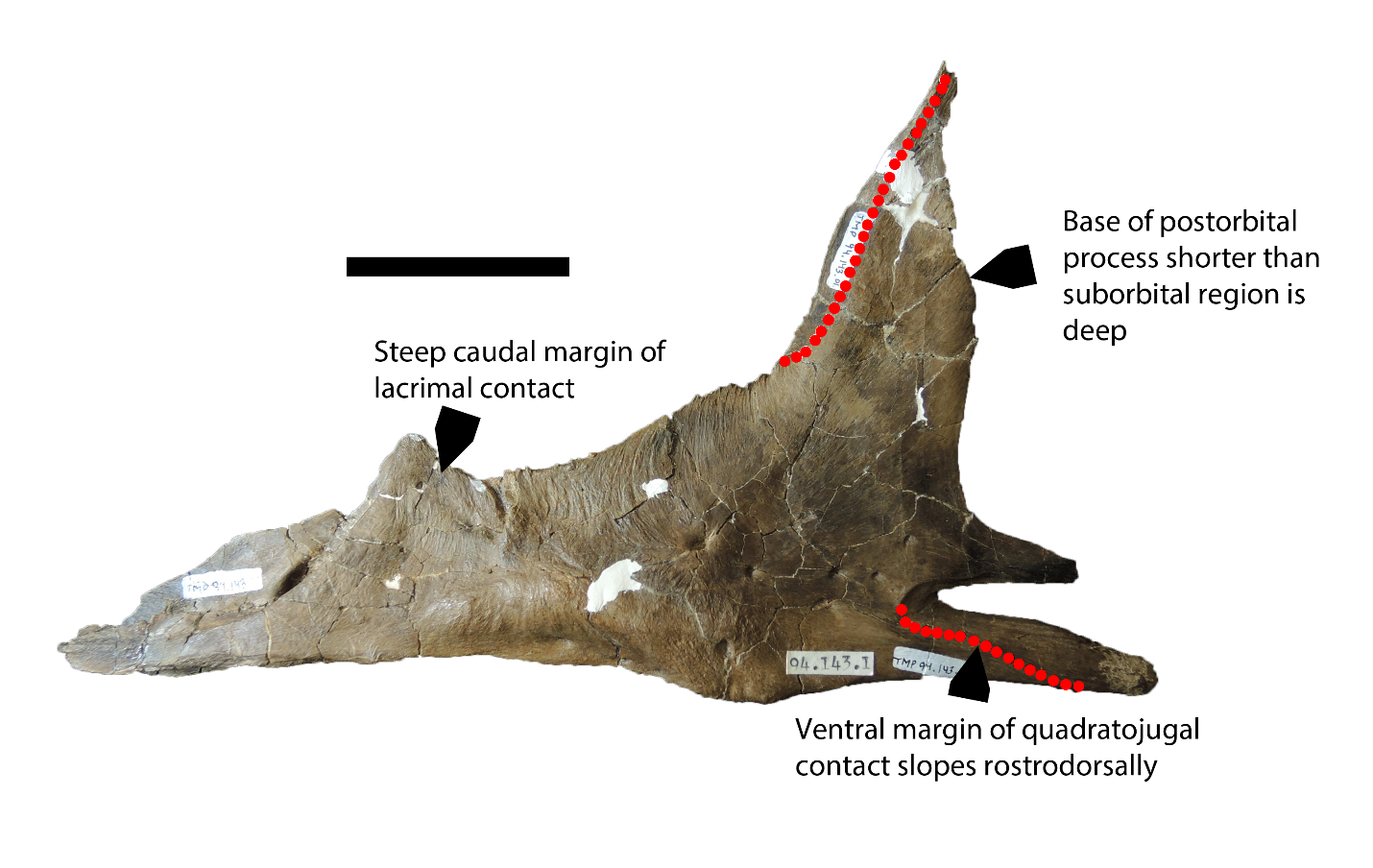
 Figure S2**. Left jugal of TMP 1994.143.1 in lateral view. Dotted lines indicate contact margin of postorbital (dorsal) and quadratojugal (posteroventral) articulations.


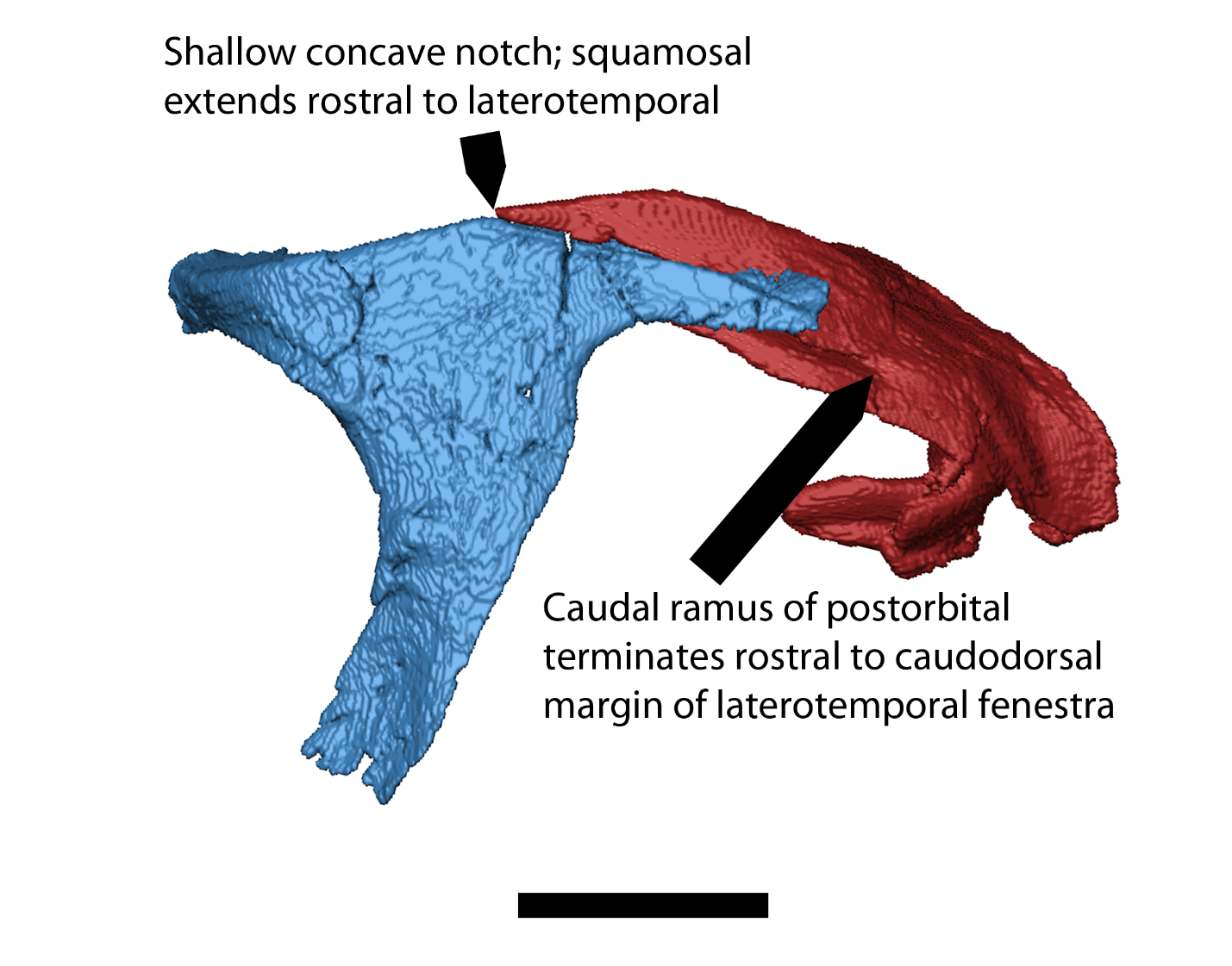


**Figure S3.** Left postorbital (blue) and squamosal (red) of TMP 1994.143.1 in articulation. Scale bar equals 50 mm.


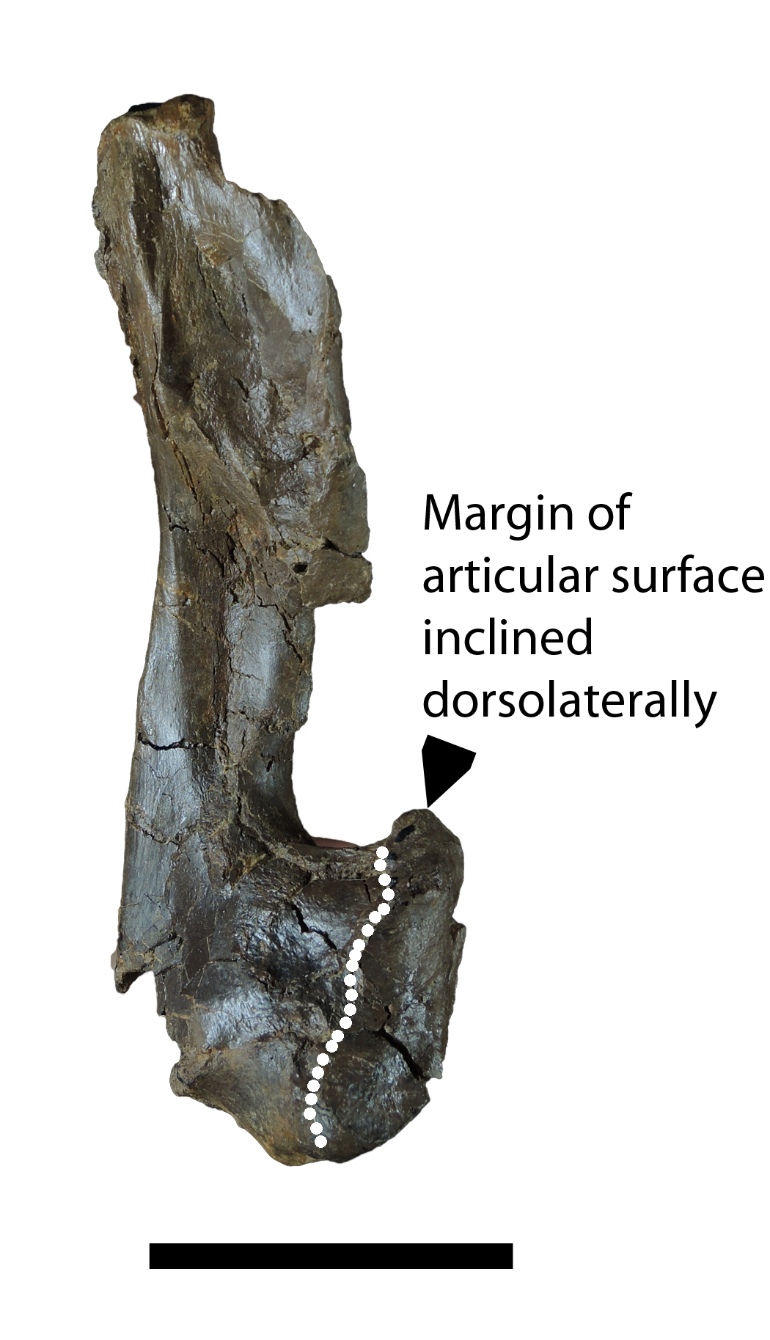


**Figure S4.** Right quadrate of TMP 1994.143.1 in posterior view. Dotted line indicates medial margin of quadratojugal contact on the lateral condyle. Scale bar equals 50 mm.


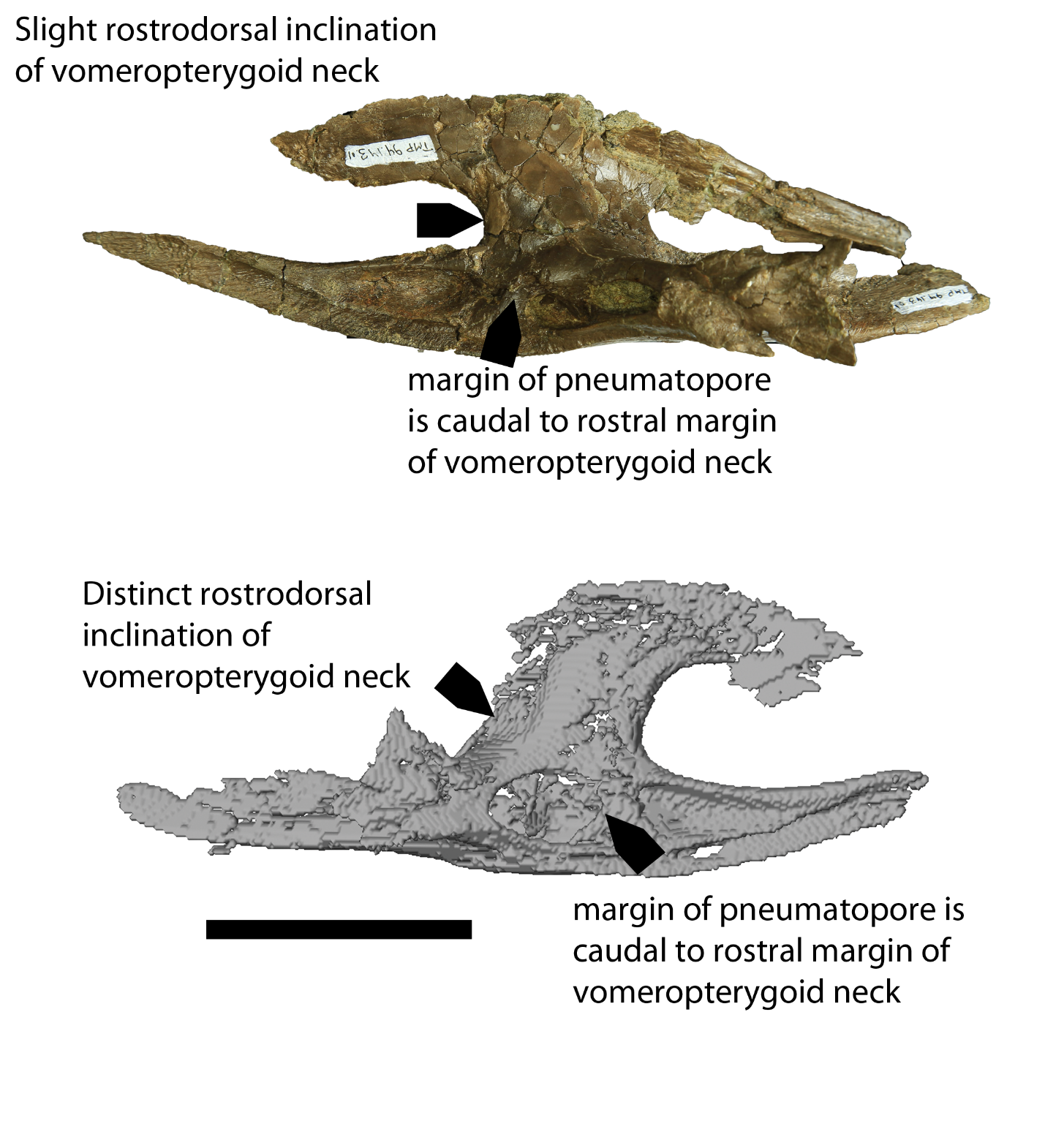


**Figure S5.** Left palatine of TMP 1994.143.1 (top) and digital rendering (based on CT data) of right palatine of juvenile *Gorgosaurus* specimen TMP 2009.12.14, both in lateral view. Scale bar equals 50 mm.


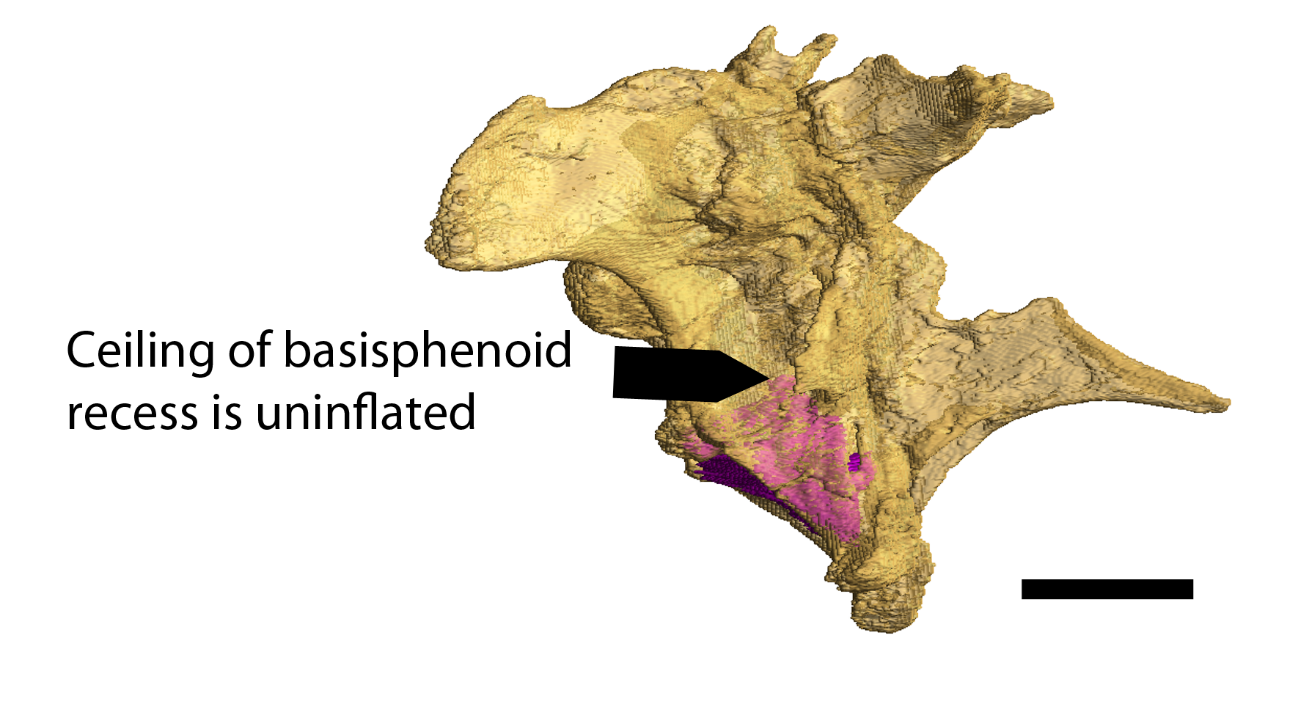


**Figure S6.** Digital rendering (based on CT data) of the braincase of TMP 1994.143.1 in lateral view. Scale bar equals 50 mm.


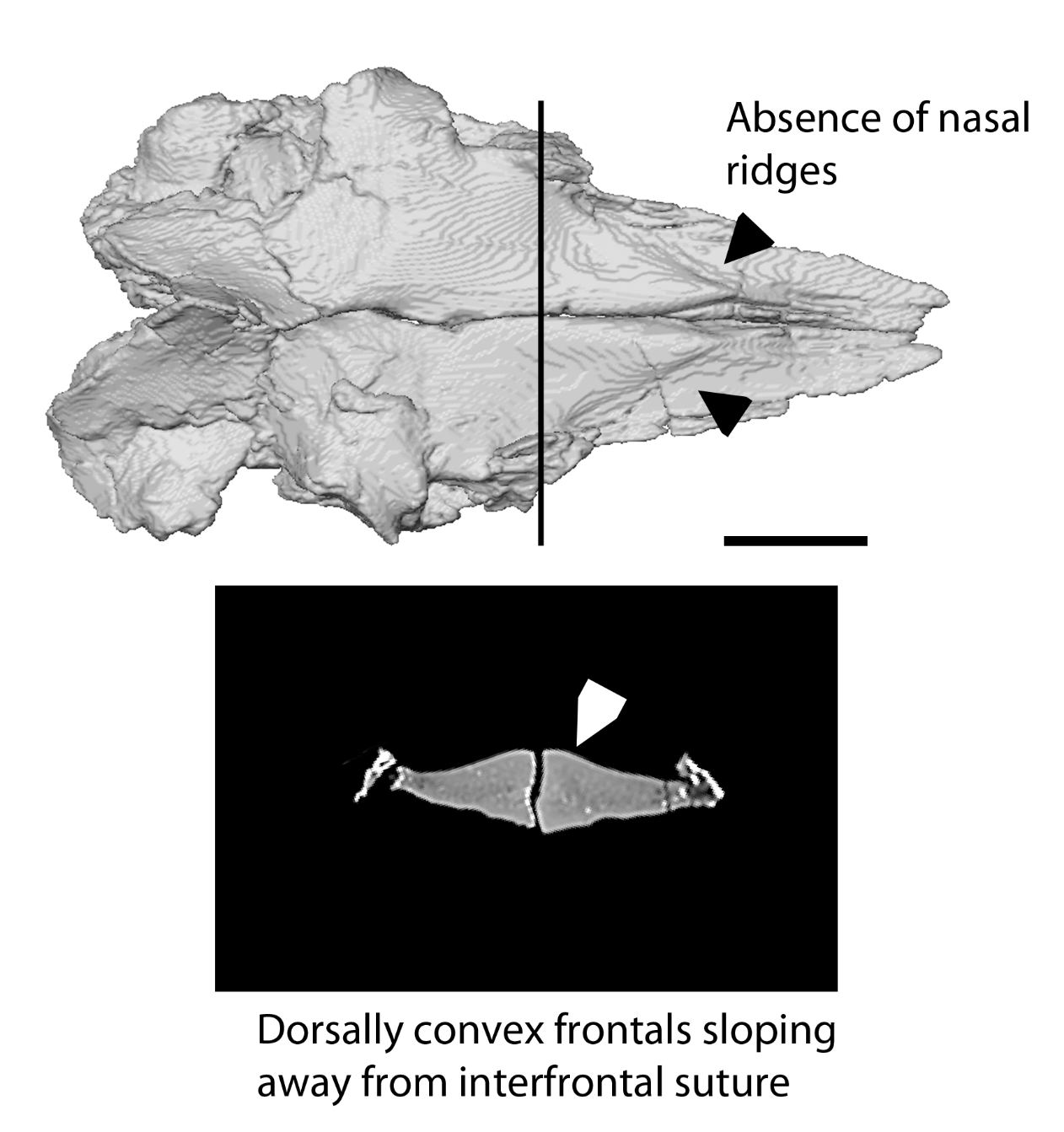


**Figure S7.** Frontals of TMP 1994.143.1 in dorsal view (top) and CT cross section (bottom) through marked plane in top image. Scale bar equals 50 mm.


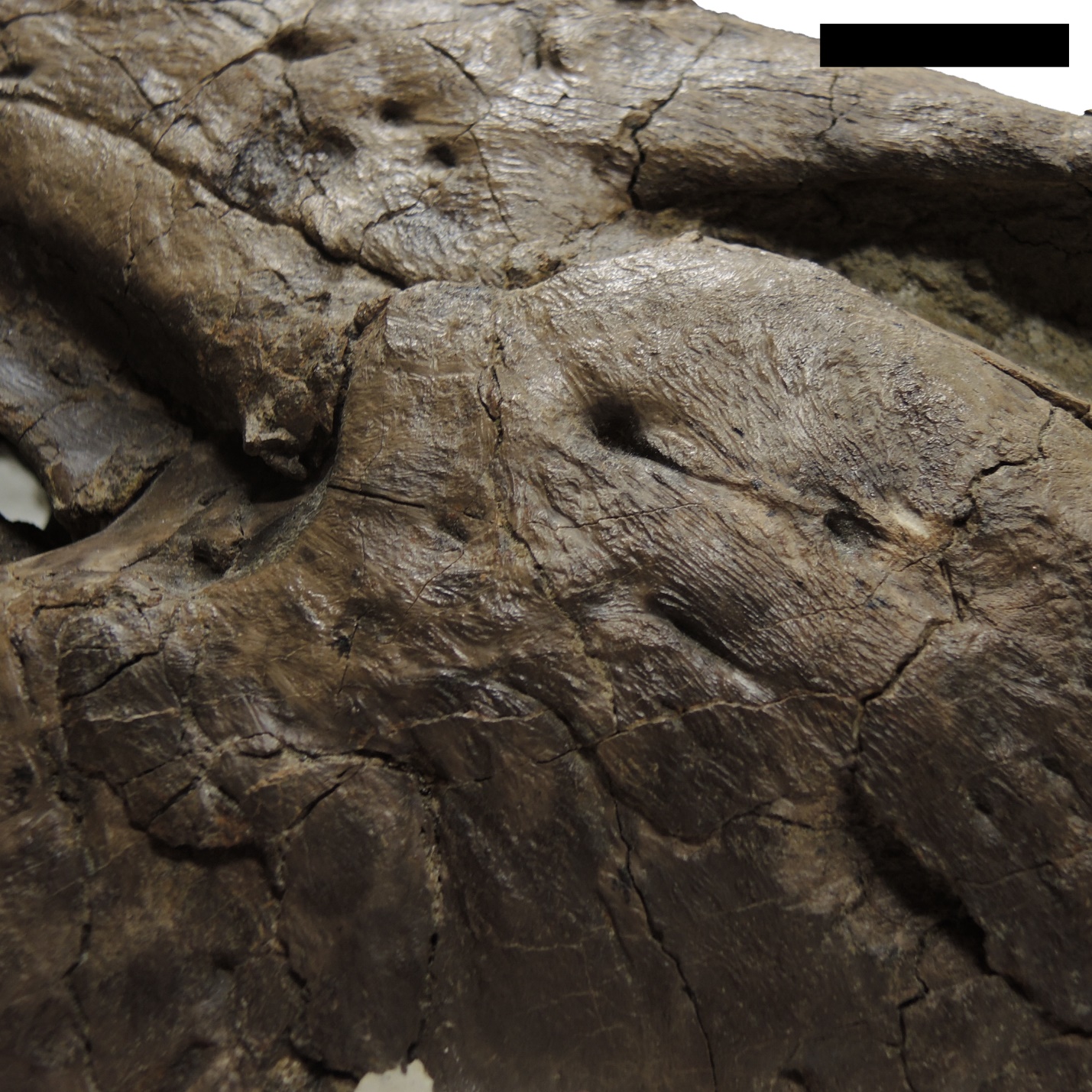


**Figure S8.** Subcutaneous surface of maxilla of TMP 1994.143.1 showing the absence of deep and distinct sulci diagnostic of *Daspletosaurus*. The presence of low ridges and shallow grooves is, however, typical of albertosaurines. Scale bar equals 20 mm.


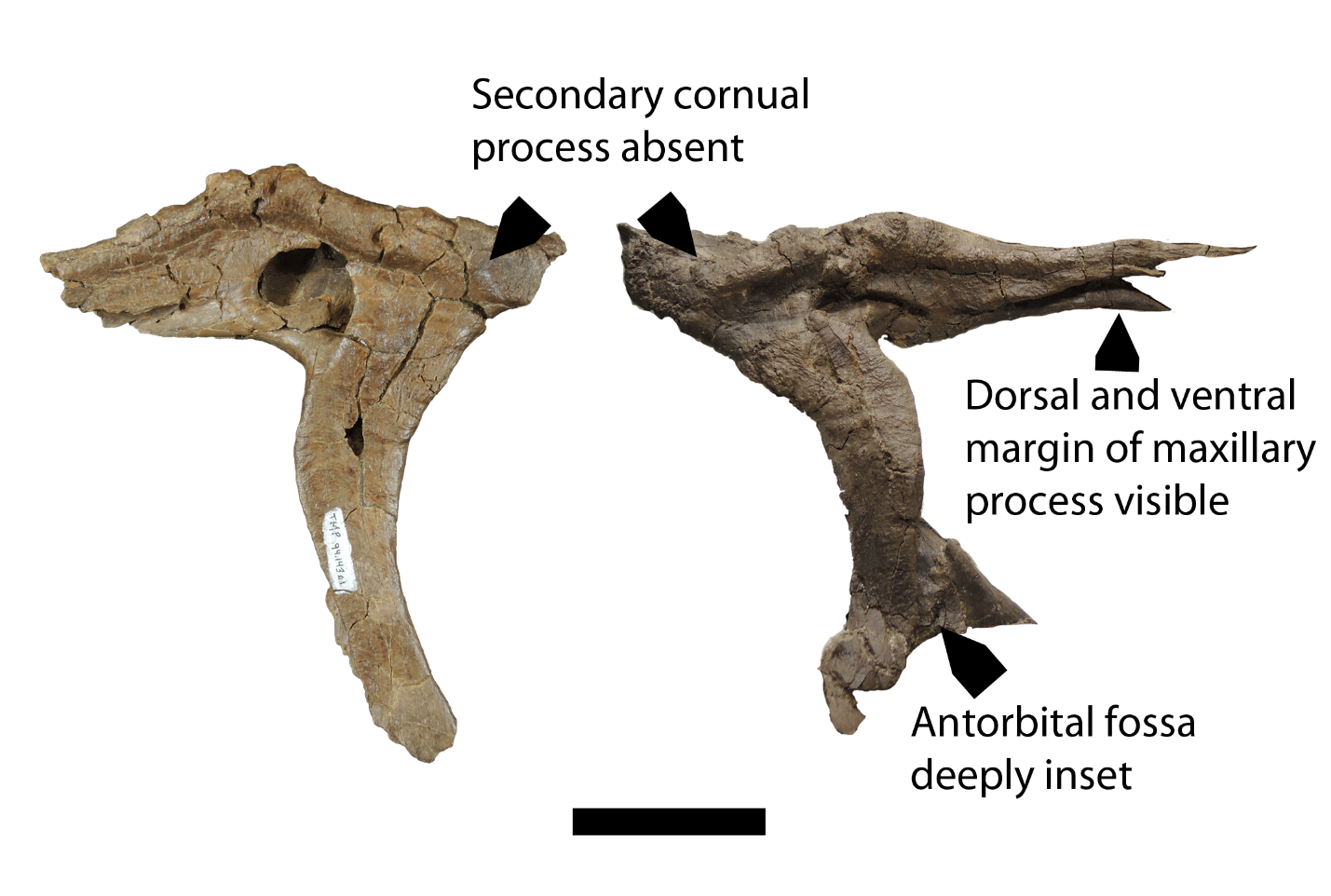


**Figure S9.** Left and right, respectively, lacrimals of TMP 1994.143.1 in lateral view. Scale bar equals 50 mm.


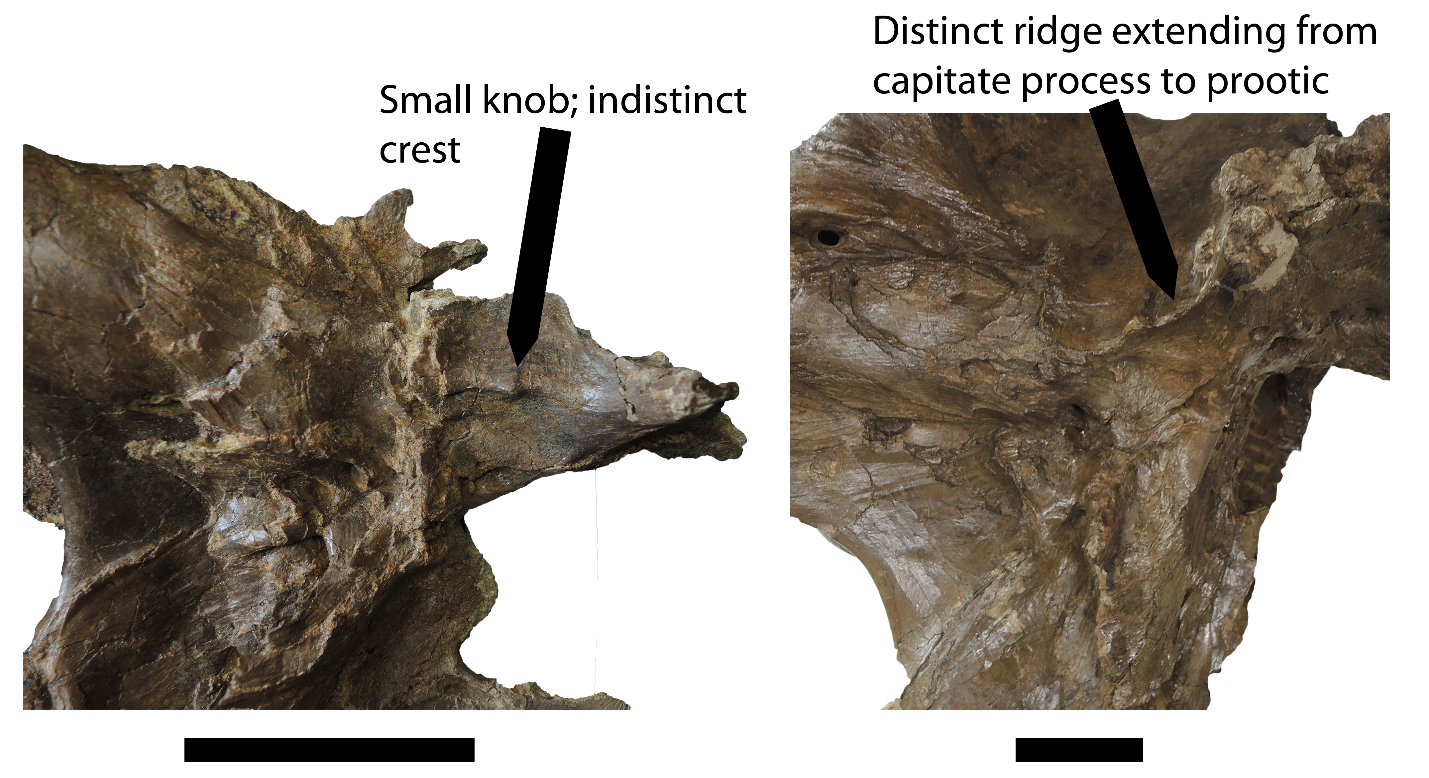


**Figure S10.** Braincase of TMP 1994.143.1 (left) and *Daspletosaurus* holotype CMN 8506 (right). Scale bar equals 100 mm.


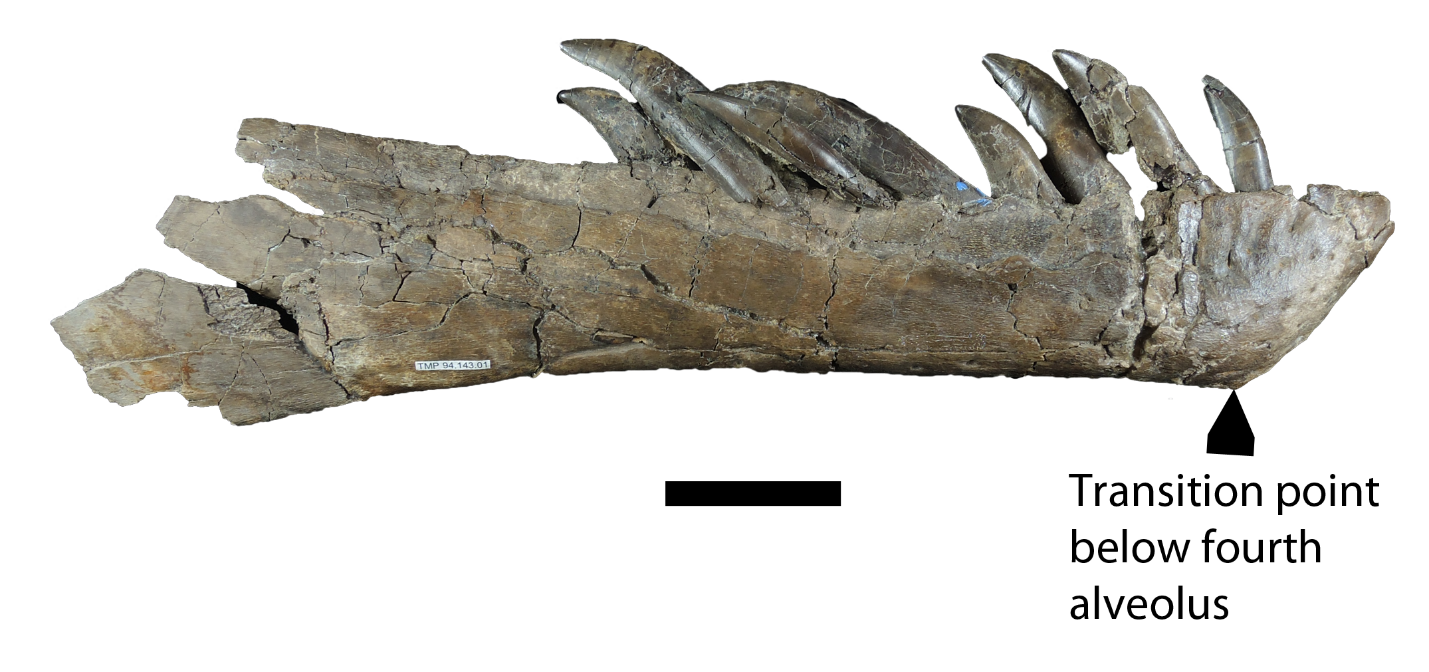


**Figure S11.** Right dentary of TMP 1994.143.1 in lateral view. Scale bar equals 50 mm.


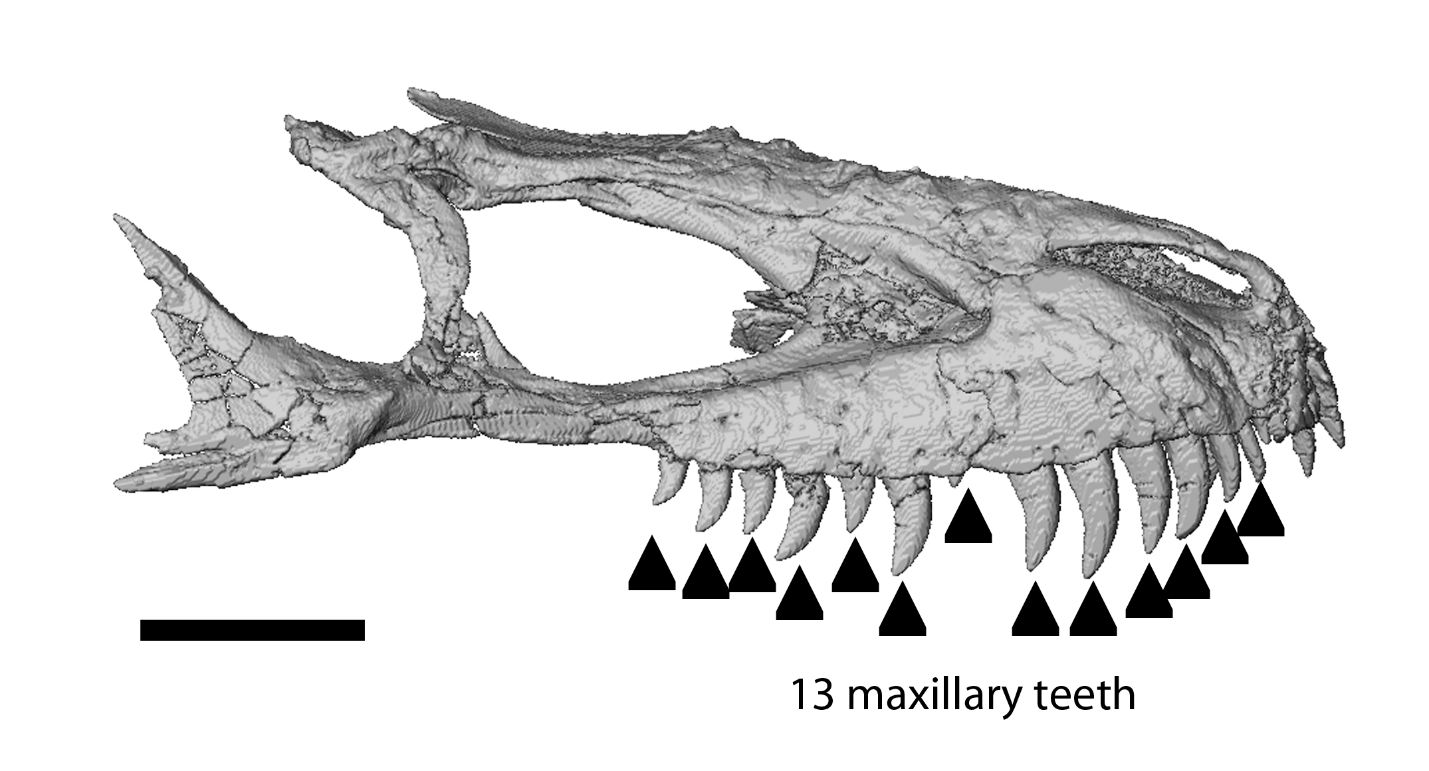


**Figure S12.** Right side of articulated rostrum of TMP 1994.143.1 with arrows denoting tooth positions. Scale bar equals 100 mm.





**Figure S13.** Left and right squamosals of TMP 1994.143.1 in dorsal view. The ridge delimiting the lateral margin of the dorsotemporal fossa is undivided in the left element but partially divided by a short and shallow groove in the right. Because this groove is neither anteroposteriorly extensive nor separates the ridge into two distinct ridges, the condition in TMP 1994.143.1 is interpreted as individual variation. Scale bar equals 50 mm.


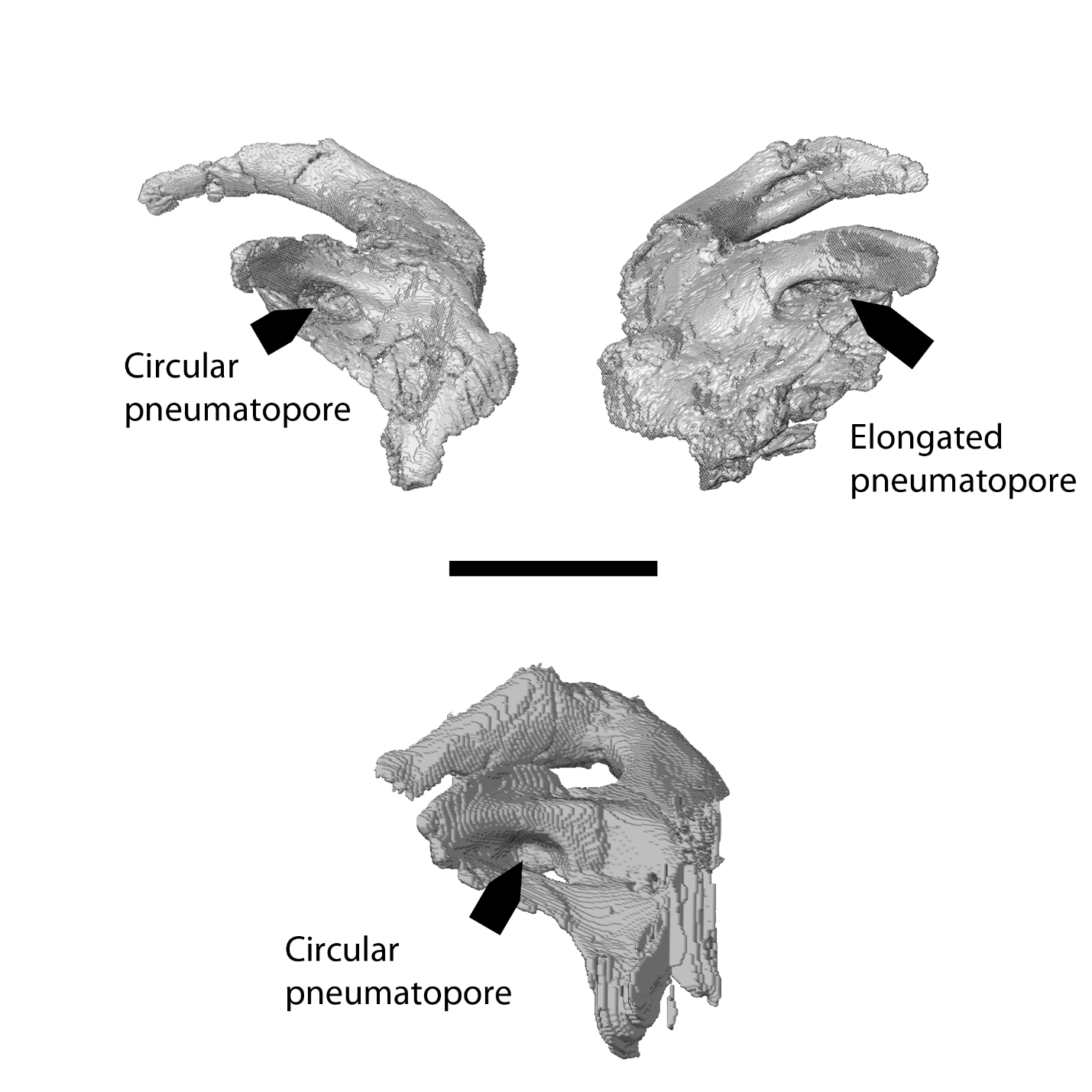


**Figure S14.** Left and right ectopterygoids of TMP 1994.143.1 (top) and left ectopterygoid of large juvenile *Gorgosaurus* individual TMP 1991.36.500 (bottom) in ventral view. The presence of a circular pneumatopore in TMP 1991.36.500 indicates the feature is not exclusive to tyrannosaurines. In addition, the left ectopterygoid of TMP 1994.143.1 is pathological which may factor into the presence of a circular pneumatopore therein. Scale bar equals 50 mm.


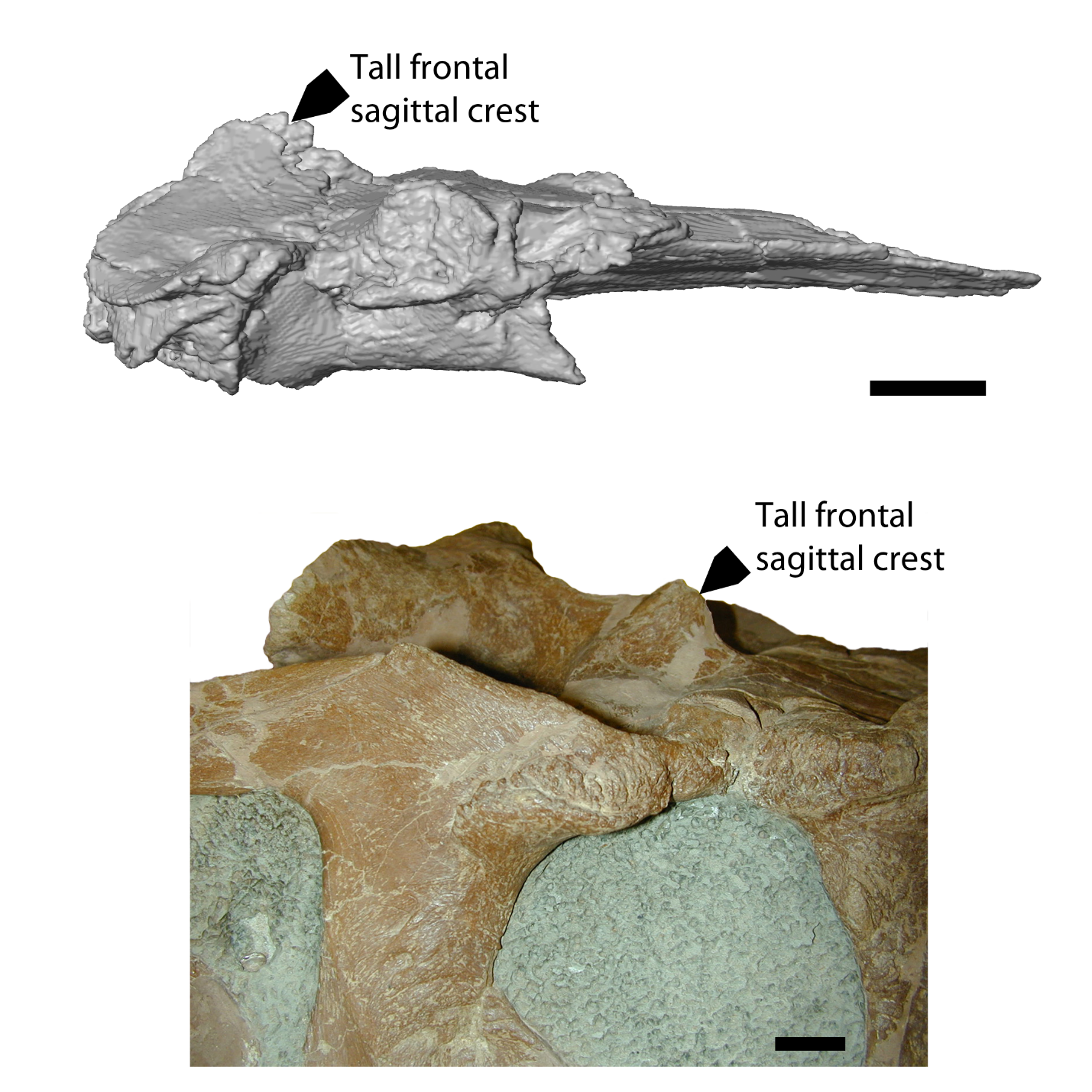


**Figure S15.** Digitally articulated frontals of TMP 1994.1431 (top) and temporal region of large *Gorgosaurus* individual UALVP10 skull (bottom) in lateral view. The increased height of the frontal sagittal crest in TMP 1994.143.1 may be due to taphonomic mediolateral compression or individual variation as some other *Gorgosaurus* specimens, such as UALVP 10, also possess sagittal crests taller than typical of albertosaurines. Scale bars equal 20 mm.

**References**

1. Carr, T. D., Varricchio, D. J., Sedlmayr, J. C., Roberts, E. M., & Moore, J. R. A new tyrannosaur with evidence for anagenesis and crocodile-like facial sensory system. *Sci. Rep.* **7**, 1–11 (2017).
